# methylKit: a comprehensive R package for the analysis of genome-wide DNA methylation profiles

**DOI:** 10.1186/gb-2012-13-10-r87

**Published:** 2012-10-03

**Authors:** Altuna Akalin, Matthias Kormaksson, Sheng Li, Francine E Garrett-Bakelman, Maria E Figueroa, Ari Melnick, Christopher E Mason

**Affiliations:** 1 Department of Physiology and Biophysics, 1305 York Ave., Weill Cornell Medical College, New York, NY 10065, USA; 2 The HRH Prince Alwaleed Bin Talal Bin Abdulaziz Alsaud Institute for Computational Biomedicine, 1305 York Ave., Weill Cornell Medical College, New York, NY 10065, USA; 3 Department of Public Health, Weill Cornell Medical College, 1300 York Ave., New York, NY 10065, USA; 4 Department of Medicine, Division of Hematology/Oncology, 1300 York Ave., Weill Cornell Medical College, New York, NY 10065, USA; 5 Department of Pathology, University of Michigan, 109 Zina Pitcher Place, Ann Arbor, MI 48109, USA; 6 Department of Pharmacology, 1300 York Ave., Weill Cornell Medical College, New York, NY 10065, USA

## Abstract

DNA methylation is a chemical modification of cytosine bases that is pivotal for gene regulation, cellular specification and cancer development. Here, we describe an R package, methylKit, that rapidly analyzes genome-wide cytosine epigenetic profiles from high-throughput methylation and hydroxymethylation sequencing experiments. methylKit includes functions for clustering, sample quality visualization, differential methylation analysis and annotation features, thus automating and simplifying many of the steps for discerning statistically significant bases or regions of DNA methylation. Finally, we demonstrate methylKit on breast cancer data, in which we find statistically significant regions of differential methylation and stratify tumor subtypes. methylKit is available at http://code.google.com/p/methylkit.

## Rationale

DNA methylation is a critical epigenetic modification that guides development, cellular differentiation and the manifestation of some cancers [1,2]. Specifically, cytosine methylation is a widespread modification in the genome, and it most often occurs in CpG dinucleotides, although non-CpG cytosines are also methylated in certain tissues such as embryonic stem cells [3]. DNA methylation is one of the many epigenetic control mechanisms associated with gene regulation. Specifically, cytosine methylation can directly hinder binding of transcription factors and methylated bases can also be bound by methyl-binding-domain proteins that recruit chromatin-remodeling factors [4,5]. In addition, aberrant DNA methylation patterns have been observed in many human malignancies and can also be used to define the severity of leukemia subtypes [6]. In malignant tissues, DNA is either hypo-methylated or hyper-methylated compared to the normal tissue. The location of hyper- and hypo-methylated sites gives distinct signatures within many diseases [7]. Often, hypomethylation is associated with gene activation and hypermethylation is associated with gene repression, although there are many exceptions to this trend [7]. DNA methylation is also involved in genomic imprinting, where the methylation state of a gene is inherited from the parents, but *de novo *methylation also can occur in the early stages of development [8,9].

A common technique for measuring DNA methylation is bisulfite sequencing, which has the advantage of providing single-base, quantitative cytosine methylation levels. In this technique, DNA is treated with sodium bisulfite, which deaminates cytosine residues to uracil, but leaves 5-methylcytosine residues unaffected. Single-base resolution, %methylation levels are then calculated by counting the ratio of C/(C+T) at each base. There are multiple techniques that leverage high-throughput bisulfite sequencing such as: reduced representation bisulfite sequencing (RRBS)[10] and its variants [11], whole-genome shotgun bisulfite sequencing (BS-seq) [12], methylC-Seq [13], and target capture bisulfite sequencing [14]. In addition, 5-hydroxymethylcytosine (5hmC) levels can be measured through a modification of bisulfite sequencing techniques [15].

**Figure 1:**
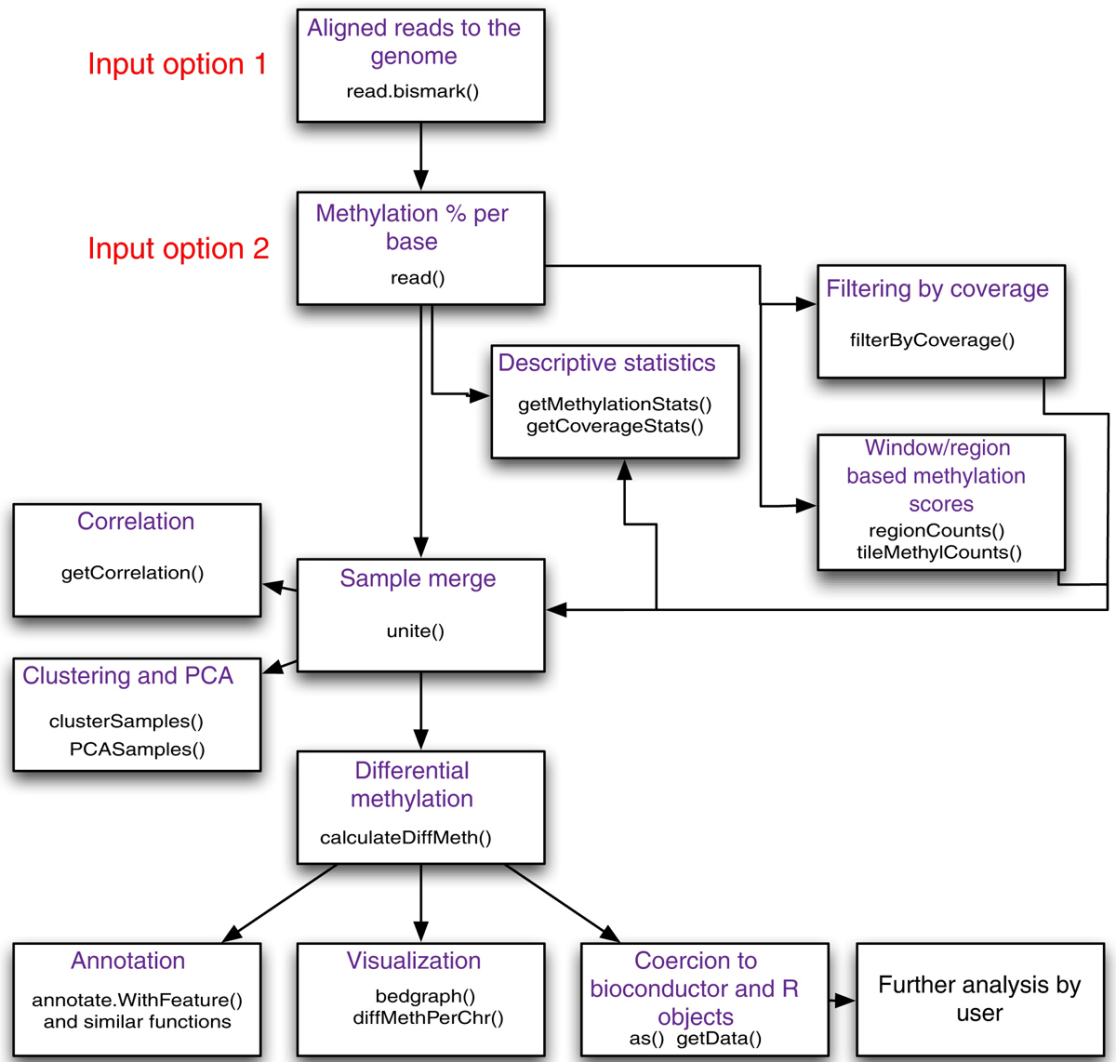
**Flowchart of possible operations by methylKit**. A summary of the most important *methylKit *features is shown in a flow chart. It depicts the main features of *methylKit *and the sequential relationship between them. The functions that could be used for those features are also printed in the boxes.

Yet, as bisulfite sequencing techniques have expanded, there are few computational tools available to analyze the data. Moreover, there is a need for an end-to-end analysis package with comprehensive features and ease of use. To address this, we have created *methylKit*, a multi-threaded R package that can rapidly analyze and characterize data from many methylation experiments at once. *methylKit *can read DNA methylation information from a text file and also from alignment files (for example, SAM files) and carry out operations such as differential methylation analysis, sample clustering and annotation, and visualization of DNA methylation events (See Figure 1 for a diagram of possible operations). *methylKit *has open-source code and is available at [16] and as Additional file 1 (see also Additional file 2 for the user guide and Additional file 3 for the package documentation ). Our data framework is also extensible to emerging methods in quantization of other base modifications, such as 5hmC [14], or sites discovered through single molecule sequencing [17,18]. For clarity, we describe only examples with DNA methylation data.

## Flexible data integration and regional analysis

High-throughput bisulfite sequencing experiments typically yield millions of reads with reduced complexity due to cytosine conversion, and there are several different aligners suited for mapping these reads to the genome (see Frith *et al*. [19] and Krueger *et al*. [20] for a review and comparison between aligners). Since *methylKit *only requires a methylation score per base for all analyses, it is a modular package that can be applied independent of any aligner. Currently, there are two ways that information can be supplied to *methylKit*:: 1) *methylKit *can read per base methylation scores from a text file (see Table 1 for an example of such a file); and, 2) *methylKit *can read SAM format [21] alignments files obtained from Bismark aligner [22]. If a SAM file is supplied, *methylkit *first processes the alignment file to get %methylation scores and then reads that information into memory.

**Table 1:** Sample text file that can be read by methylKit.

chrBase	chr	base	strand	coverage	freqC	freqT
chr21.9764539	chr21	9764539	R	12	25	75
chr21.9764513	chr21	9764513	R	12	0	100
chr21.9820622	chr21	9820622	F	13	0	100
chr21.9837545	chr21	9837545	F	11	0	100
chr21.9849022	chr21	9849022	F	124	72.58	27.42
chr21.9853326	chr21	9853326	F	17	70.59	29.41

*methylKit *can read tab-delimited text files with the following format: the text file should include a unique.id, chromosome name, base position, strand, read coverage, % of C bases and % of T bases on that location.

Most bisulfite experiments have a set of test and control samples or samples across multiple conditions, and *methylKit *can read and store (in memory) methylation data simultaneously for N-experiments, limited only by memory of the node or computer. The default setting of the processing algorithm requires that there be least 10 reads covering a base and each of the bases covering the genomic base position have at least 20 PHRED quality score. Also, since DNA methylation can occur in CpG, CHG and CHH contexts (H = A, T, or C) [3], users of *methylKit *have the option to provide methylation information for all these contexts: CpG, CHG and CHH from SAM files.

### Summarizing DNA methylation information over pre-defined regions or tiling windows

Although base-pair resolution DNA methylation information is obtained through most bisulfite sequencing experiments, it might be desirable to summarize methylation information over tiling windows or over a set of predefined regions (promoters, CpG islands, introns, and so on). For example, Smith *et al*. [9] investigated methylation profiles with RRBS experiments on gametes and zygote and summarized methylation information on 100bp tiles across the genome. Their analysis revealed a unique set of differentially methylated regions maintained in early embryo. Using tiling windows or predefined regions, such as promoters or CpG islands, is desirable when there is not enough coverage, when bases in close proximity will have similar methylation profiles, or where methylation properties of a region as a whole determines its function. In accordance with these potential analytic foci, *methylKit *provides functionality to do either analysis on tiling windows across the genome or predefined regions of the genome. After reading the base pair methylation information, users can summarize the methylation information on pre-defined regions they select or on tiling windows covering the genome (parameter for tiles are user provided). Then, subsequent analyses, such as clustering or differential methylation analysis, can be carried out with the same functions that are used for base pair resolution analysis.

### Example methylation data set: breast cancer cell lines

We demonstrated the capabilities of *methylKit *using an example data set from seven breast cancer cell lines from Sun *et al*. [23]. Four of the cell lines express estrogen receptor-alpha (MCF7, T47D, BT474, ZR75-1), and from here on are referred to as ER+. The other three cell lines (BT20, MDA-MB-231, MDA-MB-468) do not express estrogen receptor-alpha, and from here on are referred to as ER-. It has been previously shown that ER+ and ER- tumor samples have divergent gene expression profiles and that those profiles are associated with disease outcome [24,25]. Methylation profiles of these cell lines were measured using reduced RRBS [10]. The R objects contained the methylation information for breast cancer cell lines and functions that produce plots and other results that are shown in the remainder of this manuscript are in Additional file 4.

## Whole methylome characterization: descriptive statistics, sample correlation and clustering

### Descriptive statistics on DNA methylation profiles

**Figure 2:**
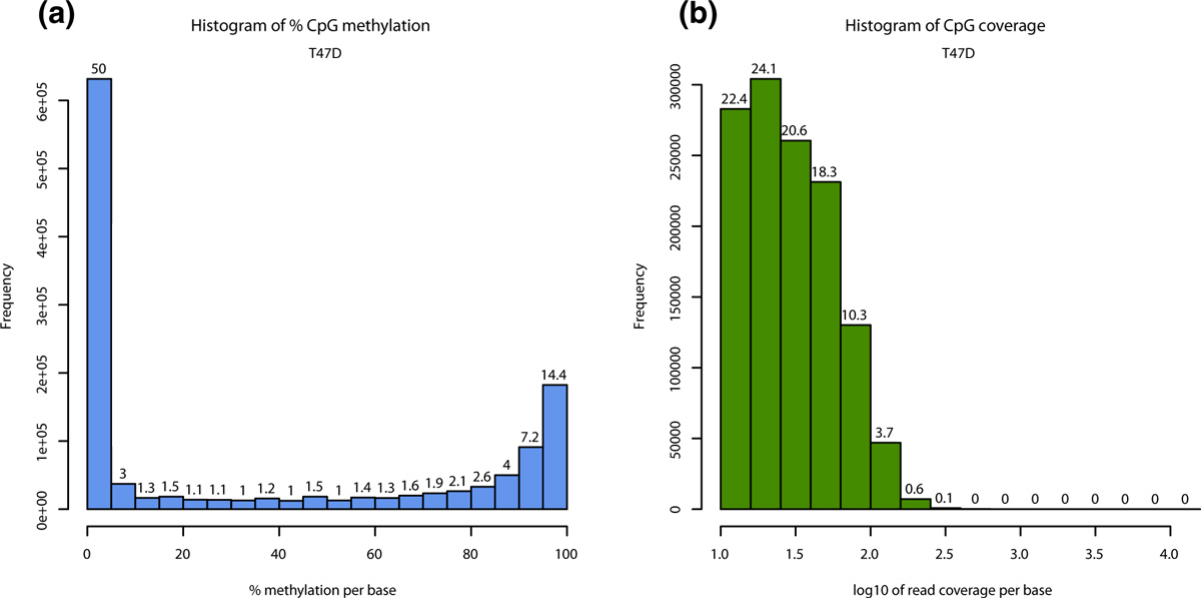
**Descriptive statistics per sample**. **(a**) Histogram of %methylation per cytosine for ER+ T47D sample. Most of the bases have either high or low methylation. (**b**) Histogram of read coverage per cytosine for ER+ T47D sample. ER+, estrogen receptor-alpha expressing.

Read coverage per base and % methylation per base are the basic information contained in the *methylKit *data structures. *methylKit *has functions for easy visualization of such information (Figure 2a and 2b for % methylation and read coverage distributions, respectively - for code see Additional file 4). In normal cells, % methylation will have a bimodal distribution, which denotes that the majority of bases have either high or low methylation. The read coverage distribution is also an important metric that will help reveal if experiments suffer from PCR duplication bias (clonal reads). If such bias occurs, some reads will be asymmetrically amplified and this will impair accurate determination of % methylation scores for those regions. If there is a high degree of PCR duplication bias, read coverage distribution will have a secondary peak on the right side. To correct for this issue, *methylKit *has the option to filter bases with very high read coverage.

### Measuring and visualizing similarity between samples

**Figure 3:**
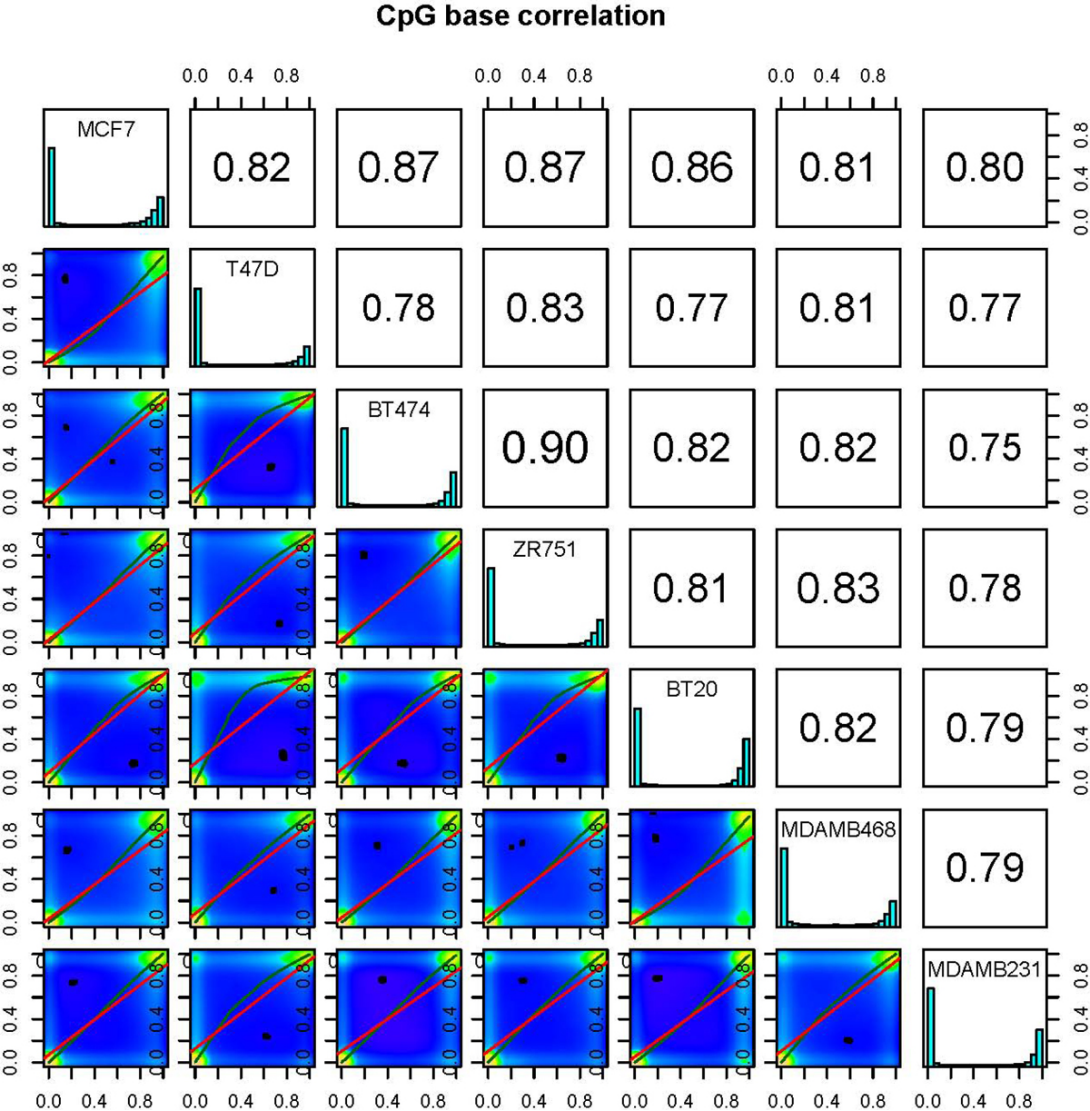
**Scatter plots for sample pairs**. Scatter plots of %methylation values for each pair in seven breast cancer cell lines. Numbers on upper right corner denote pair-wise Pearson's correlation scores. The histograms on the diagonal are %methylation histograms similar to Figure 2a for each sample.

We have also included methods to assess sample similarity. Users can calculate pairwise correlation coefficients (Pearson, Kendall or Spearman) between the %methylation profiles across all samples. However, to ensure comparable statistics, a new data structure is formed before these calculations, wherein only cytosines covered in all samples are stored. Subsequently, pairwise correlations are calculated, to produce a correlation matrix. This matrix allows the user to easily compare correlation coefficients between pairs of samples and can also be used to perform hierarchical clustering using 1- correlation distance. *methylKit *can also further visualize similarities between all pairs of samples by creating scatterplots of the %methylation scores (Figure 3). These functions are essential for detecting sample outliers or for functional clustering of samples based on their molecular signatures.

### Hierarchical clustering of samples

**Figure 4:**
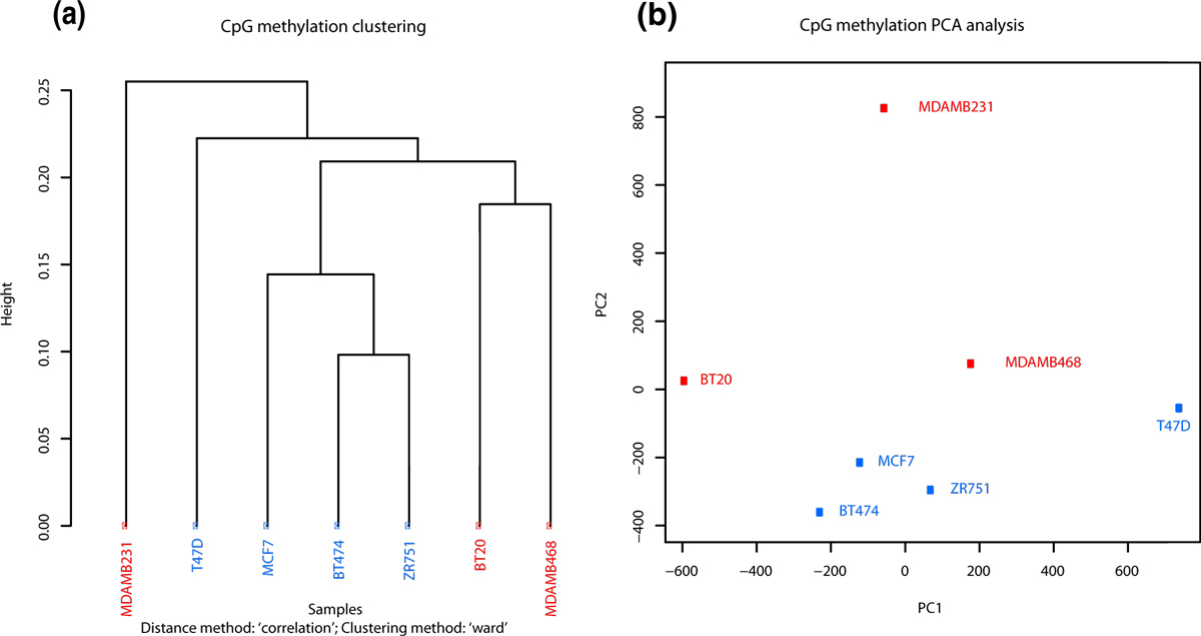
**Sample clustering**. (**a**) Hierarchical clustering of seven breast cancer methylation profiles using 1-Pearson's correlation distance. (b) Principal Component Analysis (PCA) of seven breast cancer methylation profiles, plot shows principal component 1 and principal component 2 for each sample. Samples closer to each other in principal component space are similar in their methylation profiles.

*methylKit *can also be used to cluster samples hierarchically in a variety of ways. The user can specify the distance metric between samples ('1 - correlation' 'Euclidean', 'maximum', 'manhattan', 'canberra', 'binary' or 'minkowski') as well as the agglomeration method to be used in the hierarchical clustering algorithm (for example, 'Ward's method', or 'single/complete linkage', and so on). Results can either be returned as a dendrogram object or a plot. Dendrogram plots will be color coded based on user defined groupings of samples. For example, we found that most ER+ and ER- samples clustered together except MDMB231 (Figure 4a). Moreover, the user may be interested in employing other more model-intensive clustering algorithms to their data. Users can easily obtain the %methylation data from *methylKit *object and perform their own analysis with the multitude of R-packages already available for clustering. An example of such a procedure (k-means clustering) is shown in Additional file 4.

### Principal component analysis of samples

*methylKit *can be used to perform Principal Component Analysis (PCA) on the samples' %-methylation profiles (see for example [26]). PCA can reduce the high dimensionality of a data set by transforming the large number of regions to a few principal components. The principal components are ordered so that the first few retain most of the variation present in the original data and are often used to emphasize grouping structure in the data. For example, a plot of the first two or three principal components could potentially reveal a biologically meaningful clustering of the samples. Before the PCA is performed, a new data matrix is formed, containing the samples and only those cytosines that are covered in all samples. After PCA, *methylKit *then returns to the user a 'prcomp' object, which can be used to extract and plot the principal components. We found that in the breast cancer data set, PCA reveals a similar clustering to the hierarchical clustering where MDMB231 is an outlier.

## Differential methylation calculation

### Parallelized methods for detecting significant methylation changes

Differential methylation patterns have been previously described in malignancies [27-29] and can be used to differentiate cancer and normal cells [30]. In addition, normal human tissues harbor unique DNA methylation profiles [7]. Differential DNA methylation is usually calculated by comparing methylation levels between multiple conditions, which can reveal important locations of divergent changes between a test and a control set. We have designed *methylKit *to implement two main methods for determining differential methylation across all regions: logistic regression and Fisher's exact test. However, the data frames in *methylKit *can easily be used with other statistical tests and an example is shown in Additional file 4 (using a moderated t-test, although we maintain that most natural tests for this kind of data are Fisher's exact and logistic regression based tests). For our example data set we compared ER+ to ER- samples, with our 'control group' being the ER- set.

### Method #1: logistic regression

In logistic regression, information from each sample is specified (the number of methylated Cs and number of unmethylated Cs at a given region), and a logistic regression test will be applied to compare fraction of methylated Cs across the test and the control groups. More specifically, at a given base/region we model the methylation proportion P_i_, for sample i= 1,...,n (where n is the number of biological samples) through the logistic regression model:

(1) $$\text{log}({\text{P}}_{\text{i}}\text{/(1 - }{\text{P}}_{\text{i}}\text{)) = }\beta_{0}+\beta_{1}*{\text{T}}_{\text{i}}$$

where T_i _denotes the treatment indicator for sample i, T_i _= 1 if sample i is in the treatment group and T_i _= 0 if sample i is in control group. The parameter β_0 _denotes the log odds of the control group and β_1 _the log oddsratio between the treatment and control group. Therefore, independent tests for all the bases/regions of interest are against the null hypothesis H_0_: β_1_= 0. If the null hypothesis is rejected it implies that the logodds (and hence the methylation proportions) are different between the treatment and the control group and the base/region would subsequently be classified as a differentially methylated cytosine (DMC) or region (DMR). However, if the null hypothesis is not rejected it implies no statistically significant difference in methylation between the two groups. One important consideration in logistic regression is the sample size and in many biological experiments the number of biological samples in each group can be quite small. However, it is important to keep in mind that the relevant sample sizes in logistic regression are not merely the number of biological samples but rather the total read coverages summed over all samples in each group separately. For our example dataset, we used bases with at least 10 reads coverage for each biological sample and we advise (at least) the same for other users to improve power to detect DMCs/DMRs.

In addition, we have designed *methylKit *such that the logistic regression framework can be generalized to handle more than two experimental groups or data types. In such a case, the inclusion of additional treatment indicators is analogous to multiple regression when there are categorical variables with multiple groups. Additional covariates can be incorporated into model (1) by adding to the right side of the model:

$$\alpha_{1}*\text{Covariat}{\text{e}}_{\text{1,i}}+...+\alpha_{\text{K}}*\text{Covariat}{\text{e}}_{\text{K,i}}$$

where Covariate_1,i_, ..., Covariate_K,i _denote K measured covariates (continuous or categorical) for sample i = 1,...,n and α_1_,..., α_k _denote the corresponding parameters.

### Method #2: Fisher's exact test

The Fisher's exact test compares the fraction of methylated Cs in test and control samples in the absence of replicates. The main advantage of logistic regression over Fisher's exact test is that it allows for the inclusion of sample specific covariates (continuous or categorical) and the ability to adjust for confounding variables. In practice, the number of samples per group will determine which of the two methods will be used (logistic regression or Fisher's exact test). If there are multiple samples per group, *methylKit *will employ the logistic regression test. Otherwise, when there is one sample per group, Fisher's exact test will be used.

Following the differential methylation test and calculation of *P*-values, *methylKit *will use the sliding linear model (SLIM) method to correct *P*-values to q-values [31], which corrects for the problem of multiple hypothesis testing [32,33]. However, we also implemented the standard false discovery rate (FDR)-based method (Benjamini-Hochberg) as an option for *P*-value correction, which is faster but more conservative. Finally, *methylKit *can use multi-threading so that differential methylation calculations can be parallelized over multiple cores and be completed faster.

### Extraction and visualization of differential methylation events

We have designed *methylKit *to allow a user to specify the parameters that define the DMCs/DMRs based on: q-value, %methylation difference, and type of differential methylation (hypo-/hyper-). By default, it will extract bases/regions with a q-value <0.01 and %methylation difference >25%. These defaults can easily be changed when calling *get.methylDiff() *function. In addition, users can specify if they want hyper-methylated bases/regions (bases/regions with higher methylation compared to control samples) or hypo-methylated bases/regions (bases/regions with lower methylation compared to control samples). In the literature, hyper- or hypo-methylated DMCs/DMRs are usually defined relative to a control group. In our examples, and in *methylKit *in general, a control group is defined when creating the objects through supplied treatment vector, and hyper-/hypomethylation definitions are based on that control group.

**Figure 5:**
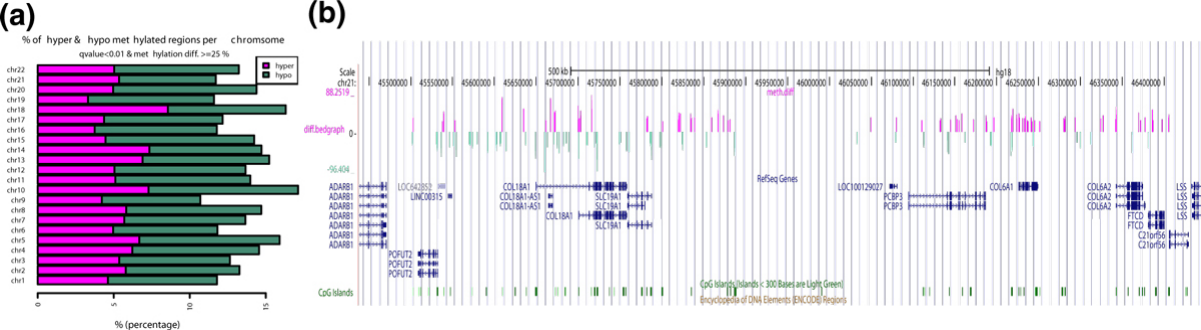
**Visualizing differential methylation events**. (**a**) Horizontal bar plots show the number of hyper- and hypomethylation events per chromosome, as a percent of the sites with the minimum coverage and differential. By default this is a 25% change in methylation and all samples with 10X coverage. (**b**) Example of bedgraph file uploaded to UCSC browser. The bedraph file is for differentially methylated CpGs with at least a 25% difference and q-value <0.01. Hyper- and hypo-methylated bases are color coded. The bar heights correspond to % methylation difference between ER+ and ER- sets. ER+, estrogen receptor-alpha expressing; ER-, estrogen receptor-alpha non-expressing. UCSC, University of California Santa Cruz.

Furthermore, DMCs/DMRs can be visualized as horizontal barplots showing percentage of hyper- and hypo-methylated bases/regions out of covered cytosines over all chromosomes (Figure 5a). We observed higher levels of hypomethylation than hypermethylation in the breast cancer cell lines, which indicates that ER+ cells have lower levels of methylation. Since another common way to visualize differential methylation events is with a genome browser, *methylKit *can output bedgraph tracks (Figure 5b) for use with the UCSC Genome Browser or Integrated Genome Viewer.

## Annotating differential methylation events

### Annotation with gene models and CpG islands

**Figure 6:**
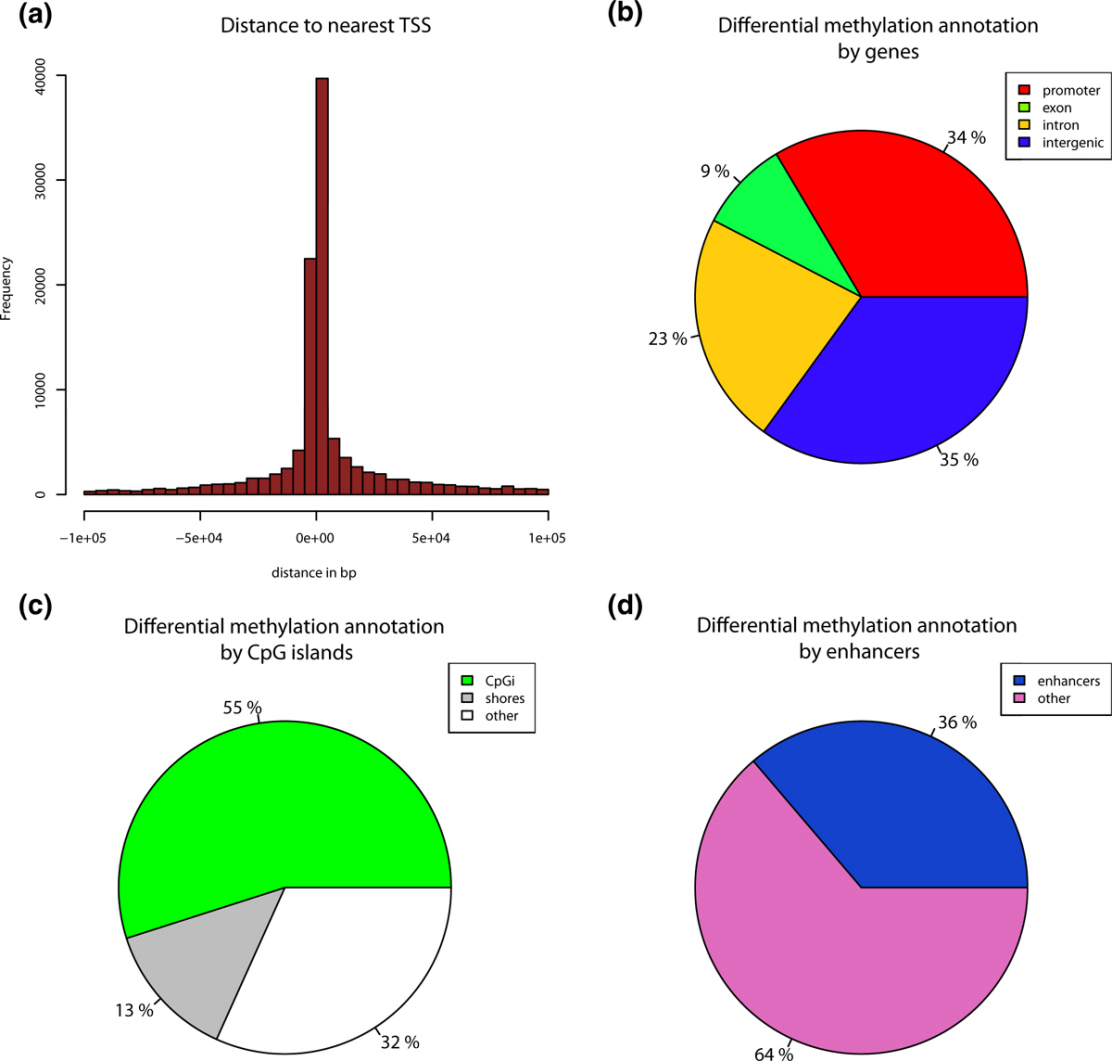
**Annotation of differentially methylated CpGs**. (**a**) Distance to TSS for differentially methylated CpGs are plotted from ER+ versus ER- analysis. (**b**) Pie chart showing percentages of differentially methylated CpGs on promoters, exons, introns and intergenic regions. (**c**) Pie chart showing percentages of differentially methylated CpGs on CpG islands, CpG island shores (defined as 2kb flanks of CpG islands) and other regions outside of shores and CpG islands. (**d**) Pie chart showing percentages of differentially methylated CpGs on enhancers and other regions. ER+, estrogen receptor-alpha expressing; ER-, estrogen receptor-alpha non-expressing, TSS, transcription start site.

To discern the biological impact of differential methylation events, each event must be put into its genomic context for subsequent analysis. Indeed, Hansen *et al*. [34] showed that most variable regions in terms of methylation in the human genome are CpG island shores, rather than CpG islands themselves. Thus, it is interesting to know the location of differential methylation events with regard to CpG islands, their shores, and also the proximity to the nearest transcription start site (TSS) and gene components. Accordingly, *methylKit *can annotate differential methylation events with regard to the nearest TSS (Figure 6a) and it also can annotate regions based on their overlap with CpG islands/shores and regions within genes (Figures 6b and 6c are output from methylKit).

### Annotation with custom regions

As with most genome-wide assays, the regions of interest for DNA methylation analysis may be quite numerous. For example, several reports show that Alu elements are aberrantly methylated in cancers [35,36] and enhancers are also differentially methylated [37,38]. Since users may need to focus on specific genomic regions and require customized annotation for capturing differential DNA methylation events, *methylKit *can annotate differential methylation events using user-supplied regions. As an example, we identified differentially methylated bases of ER+ and ER- cells that overlap with ENCODE enhancer regions [39], and we found a large proportion of differentially methylated CpGs overlapping with the enhancer marks, and then plotted them with *methylKit *(Figure 6d).

## Analyzing 5-hydroxymethylcytosine data with methylKit

5-Hydroxymethylcytosine is a base modification associated with pluropotency, hematopoiesis and certain brain tissues (reviewed in [40]). It is possible to measure base-pair resolution 5hmC levels using variations of traditional bisulfite sequencing. Recently, Yu *et al*. [41] and Booth *et al*. [15] published similar methods for detecting 5hmC levels in base-pair resolution. Both methods require measuring 5hmC and 5mC levels simultaneously and use 5hmC levels as a substrate to deduce real 5mC levels, since traditional bisulfite sequencing cannot distinguish between the two [42]. However, both the 5hmC and 5mC data generated by these protocols are bisulfite sequencing based, and the alignments and text files of 5hmC levels can be used directly in *methylKit*. Furthermore, *methylKit *has an *adjust.methylC() *function to adjust 5mC levels based on 5hmC levels as described in Booth *et al*. [15].

## Customizing analysis with convenience functions

*methylKit *is dependent on Bioconductor [43] packages such as *GenomicRanges *and its objects are coercible to *GenomicRanges *objects and regular R data structures such as data frames via provided convenience functions. That means users can integrate *methylKit *objects to other Bioconductor and R packages and customize the analysis according to their needs or extend the analysis further by using other packages available in R.

## Conclusions

Methods for detecting methylation across the genome are widely used in research laboratories, and they are also a substantial component of the National Institutes of Health's (NIH's) EpiGenome roadmap and upcoming projects such as BLUEPRINT [44]. Thus, tools and techniques that enable researchers to process and utilize genome-wide methylation data in an easy and fast manner will be of critical utility.

Here, we show a large set of tools and cross-sample analysis algorithms built into *methylKit*, our open-source, multi-threaded R package that can be used for any base-level dataset of DNA methylation or base modifications, including 5hmC. We demonstrate its utility with breast cancer RRBS samples, provide test data sets, and also provide extensive documentation with the release.

## Abbreviations

5hmC: 5-hydroxymethylcytosine; 5mC: 5-methylcytosine; bp: base pair; BS-seq,:bisulfite sequencing; DMC: differentially methylated cytosine; DMR: differentially methylated region; ER: estrogen receptor alpha; FDR: false discovery rate; PCA: principal component analysis; PCR: polymerase chain reaction; RRBS: reduced representation bisulfite sequencing; SLIM: sliding linear model; TSS: transcription start site.

## Competing interests

The authors declare that they have no competing interests.

## Authors' contributions

AA designed *methylKit*, developed the first codebase, and added most features. MK designed the logistic regression based statistical test for methylKit and worked on statistical modeling and initial clustering features. SL wrote some of the features in *methylKit *and prepared plots for the manuscript. MEF, FGB and AM tested the code and provided initial data for development of *methylKit*. CEM supervised the work, tested code, and coordinated test data for validation. All authors have read and approved the manuscript for publication.

## Supplementary Material

Additional file 1 **methylKit v0.5.3**. This version of methylKit is included for archival purposes only. Please download the most recent version from [16]. Click here for file

Additional file 2 **methylKit User Guide**. A vignette file to accompany the methylKit software package; the most recent software and vignette can be downloaded at [16]. Click here for file

Additional file 3 **methylKit documentation**. Documentation for functions and classes in the methylKit software package; the most recent software and documentation can be downloaded at [16]. Click here for file

Additional file 4 **R script for example analysis**. The file contains R commands that are needed to do analysis and to produce graphs used in this manuscript. The file contains both the commands and detailed comments on how those commands can be used. An up to date version of this script will be consistently maintained at [16]. Click here for file
