# Supplementary material for: methylKit: a comprehensive R package for the analysis of genome-wide DNA methylation profiles
Source: Genome Biol. 2012 Oct 3;13(10):R87. doi: 10.1186/gb-2012-13-10-r87 (PMC3491415; doi:10.1186/gb-2012-13-10-r87)
Supplement: Additional file 2 — methylKit User Guide. A vignette file to accompany the methylKit software package; the most recent software and vignette can be downloaded at [16]. [file gb-2012-13-10-r87-S2.pdf]

methylKit: User Guide

Altuna Akalin
ala2027@med.cornell.edu

Matthias Kormaksson
mk375@cornell.edu

Sheng Li
shl2018@med.cornell.edu

April 28, 2012

Contents

1 Introduction **2**
- 1.1 DNA methylation 2
- 1.2 High-throughput bisulfite sequencing 2

2 Basics **3**
- 2.1 Reading the methylation call files 3
- 2.2 Reading the methylation calls from sorted Bismark alignments . 4
- 2.3 Descriptive statistics on samples 4
- 2.4 Filtering samples based on read coverage 6

3 Comparative analysis **7**
- 3.1 Merging samples 7
- 3.2 Sample Correlation 8
- 3.3 Clustering samples 9
- 3.4 Tiling windows analysis 12
- 3.5 Finding differentially methylated bases or regions 13
  - 3.5.1 Finding differentially methylated bases using multiple-cores 14

4 Annotating differentially methylated bases or regions **14**
- 4.1 Regional analysis 15
- 4.2 Convenience functions for annotation objects 16

5 methylKit convenience functions **18**
- 5.1 coercion 18
- 5.2 select 22
- 5.3 reorganize 23
- 5.4 percMethylation 24

6 Acknowledgements **24**

7 R session info **24**

1 Introduction

In this manual, we will show how to use the methylKit package. methylKit is an R package for analysis and annotation of DNA methylation information obtained by high-throughput bisulfite sequencing. The package is designed to deal with sequencing data from RRBS and its variants. But it can potentially handle whole-genome bisulfite sequencing data if proper input format is provided.

1.1 DNA methylation

DNA methylation in vertebrates typically occurs at CpG dinucleotides, however non-CpG Cs are also methylated in certain tissues such as embryonic stem cells. DNA Methylation can act as an epigenetic control mechanism for gene regulation. Methylation can hinder binding of transcription factors and/or methylated bases can be bound by methyl-binding-domain proteins which can recruit chromatin remodeling factors. In both cases, the transcription of the regulated gene will be effected. In addition, aberrant DNA methylation patterns have been associated with many human malignancies and can be used in a predictive manner. In malignant tissues, DNA is either hypo-methylated or hyper-methylated compared to the normal tissue. The location of hyper- and hypo-methylated sites gives a distinct signature to many diseases. Traditionally, hypo-methylation is associated with gene transcription (if it is on a regulatory region such as promoters) and hyper-methylation is associated with gene repression.

1.2 High-throughput bisulfite sequencing

Bisulfite sequencing is a technique that can determine DNA methylation patterns. The major difference from regular sequencing experiments is that, in bisulfite sequencing DNA is treated with bisulfite which converts cytosine residues to uracil, but leaves 5-methylcytosine residues unaffected. By sequencing and aligning those converted DNA fragments it is possible to call methylation status of a base. Usually, the methylation status of a base determined by a high-throughput bisulfite sequencing will not be a binary score, but it will be a percentage. The percentage simply determines how many of the bases that are aligning to a given cytosine location in the genome have actual C bases in the reads. Since bisulfite treatment leaves methylated Cs intact, that percentage will give us percent methylation score on that base. The reasons why we will not get a binary response are 1) the probable sequencing errors in high-throughput sequencing experiments 2) incomplete bisulfite conversion 3) (and a more likely scenario) is heterogeneity of samples and heterogeneity of paired chromosomes from the same sample

2 Basics

2.1 Reading the methylation call files

We start by reading in the methylation call data from bisulfite sequencing with `read` function. Reading in the data this way will return a methylRawList object which stores methylation information per sample for each covered base. The methylation call files are basically text files that contain percent methylation score per base. A typical methylation call file looks like this:

```
##         chrBase   chr    base strand coverage freqC  freqT
## 1 chr21.9764539 chr21 9764539      R       12 25.00  75.00
## 2 chr21.9764513 chr21 9764513      R       12  0.00 100.00
## 3 chr21.9820622 chr21 9820622      F       13  0.00 100.00
## 4 chr21.9837545 chr21 9837545      F       11  0.00 100.00
## 5 chr21.9849022 chr21 9849022      F      124 72.58  27.42
```

Most of the time bisulfite sequencing experiments have test and control samples. The test samples can be from a disease tissue while the control samples can be from a healthy tissue. You can read a set of methylation call files that have test/control conditions giving `treatment` vector option. For sake of subsequent analysis, file.list, sample.id and treatment option should have the same order. In the following example, first two files are have the sample ids "test1" and "test2" and as determined by treatment vector they belong to the same group. The third and fourth files have sample ids "ctrl1" and "ctrl2" and they belong to the same group as indicated by the treatment vector.

```
library(methylKit)
file.list <- list(system.file("extdata", "test1.myCpG.txt",
    package = "methylKit"), system.file("extdata",
"test2.myCpG.txt",
    package = "methylKit"), system.file("extdata",
"control1.myCpG.txt",
    package = "methylKit"), system.file("extdata",
"control2.myCpG.txt",
    package = "methylKit"))


# read the files to a methylRawList object: myobj
myobj <- read(file.list, sample.id = list("test1",
    "test2", "ctrl1", "ctrl2"), assembly = "hg18", treatment =
c(1,
    1, 0, 0), context = "CpG")
```

2.2 Reading the methylation calls from sorted Bismark alignments

Alternatively, methylation percentage calls can be calculated from sorted SAM file(s) from Bismark aligner and read-in to the memory. Bismark is a popular aligner for bisulfite sequencing reads [1]. `read.bismark` function is designed to read-in Bismark SAM files as `methylRaw` or `methylRawList` objects which store per base methylation calls. SAM files must be sorted by chromosome and read position columns, using 'sort' command in unix-like machines will accomplish such a sort easily.

The following command reads a sorted SAM file and creates a `methylRaw` object for CpG methylation.The user has the option to save the methylation call files to a folder given by `save.folder` option. The saved files can be read-in using the `read` function when needed.

```
my.methRaw <- read.bismark(location = system.file("extdata",
    "test.fastq_bismark.sorted.min.sam", package = "methylKit"),
    sample.id = "test1", assembly = "hg18", read.context = "CpG",
    save.folder = getwd())
```

It is also possible to read multiple SAM files at the same time, check `read.bismark` documentation.

2.3 Descriptive statistics on samples

Since we read the methylation data now, we can check the basic stats about the methylation data such as coverage and percent methylation. We now have a `methylRawList` object which contains methylation information per sample. The following command prints out percent methylation statistics for second sample: "test2"

```
getMethylationStats(myobj[[2]], plot = F, both.strands = F)

## methylation statistics per base
## summary:
##    Min. 1st Qu.  Median    Mean 3rd Qu.    Max.
##     0.0    20.0    82.8    63.2    94.7   100.0
## percentiles:
##     0%    10%    20%    30%    40%    50%    60%    70%
##   0.00   0.00   0.00  48.39  70.00  82.79  90.00  93.33
##    80%    90%    95%    99%  99.5%  99.9%   100%
##  96.43 100.00 100.00 100.00 100.00 100.00 100.00
##
```

The following command plots the histogram for percent methylation distribution.The figure below is the histogram and numbers on bars denote what

percentage of locations are contained in that bin. Typically, percent methylation histogram should have two peaks on both ends. In any given cell, any given base are either methylated or not. Therefore, looking at many cells should yield a similar pattern where we see lots of locations with high methylation and lots of locations with low methylation.

```
getMethylationStats(myobj[[2]], plot = T, both.strands = F)
```

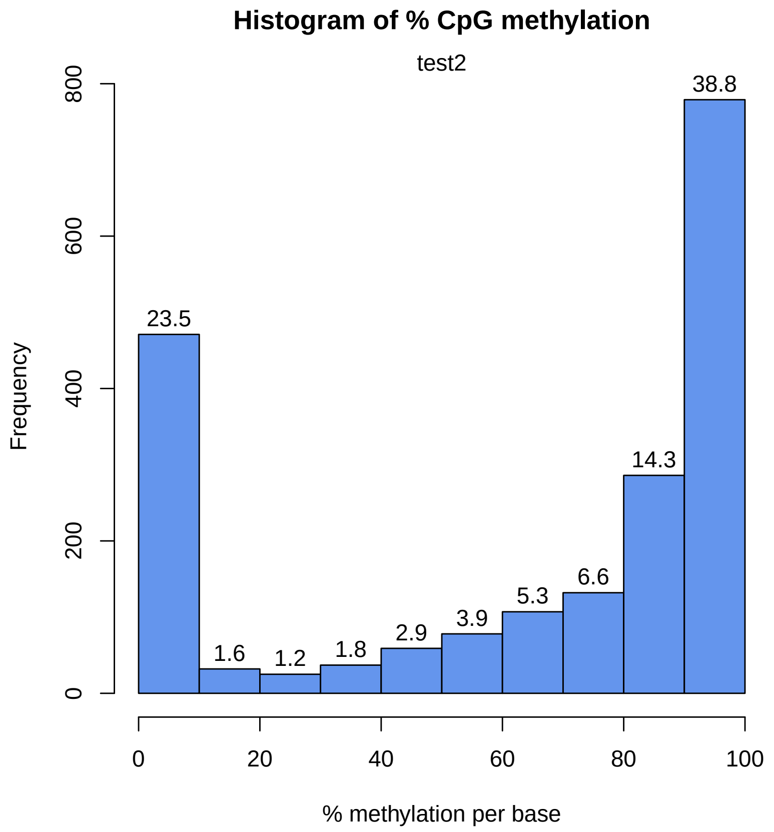

We can also plot the read coverage per base information in a similar way, again numbers on bars denote what percentage of locations are contained in that bin. Experiments that are highly suffering from PCR duplication bias will have a secondary peak towards the right hand side of the histogram.

```
library("graphics")
getCoverageStats(myobj[[2]], plot = T, both.strands = F)
```

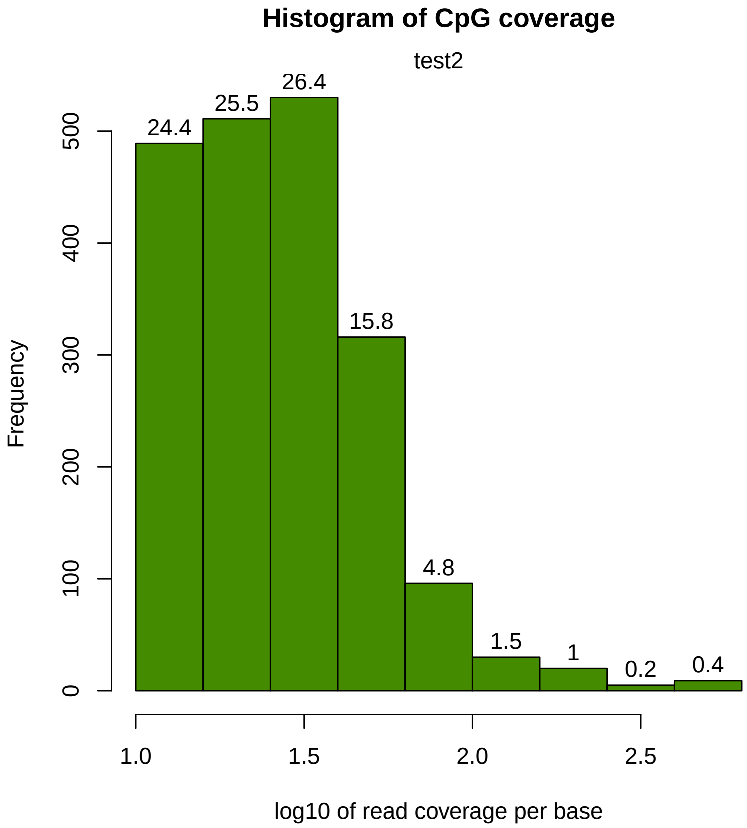

2.4 Filtering samples based on read coverage

It might be useful to filter samples based on coverage. Particularly, if our samples are suffering from PCR bias it would be useful to discard bases with very high read coverage. Furthermore, we would also like to discard bases that have low read coverage, a high enough read coverage will increase the power of the statistical tests. The code below filters a `methylRawList` and discards bases that have coverage below 10X and also discards the bases that have more than 99.9th percentile of coverage in each sample.

```
filtered.myobj <- filterByCoverage(myobj, lo.count = 10,
    lo.perc = NULL, hi.count = NULL, hi.perc = 99.9)
```

3 Comparative analysis

3.1 Merging samples

In order to do further analysis, we will need to get the bases covered in all samples. The following function will merge all samples to one object for base-pair locations that are covered in all samples. Setting `destrand`=TRUE (the default is FALSE) will merge reads on both strands of a CpG dinucleotide. This provides better coverage, but only advised when looking at CpG methylation (for CpH methylation this will cause wrong results in subsequent analyses). In addition, setting `destrand`=TRUE will only work when operating on base-pair resolution, otherwise setting this option TRUE will have no effect. The `unite()` function will return a `methylBase` object which will be our main object for all comparative analysis. The `methylBase` object contains methylation information for regions/bases that are covered in all samples.

```
meth <- unite(myobj, destrand = FALSE)
```

Let us take a look at the data content of methylBase object:

```
head(meth)

##                 id   chr    start      end strand coverage1
## 1 chr21.10011833 chr21 10011833 10011833      +       174
## 2 chr21.10011841 chr21 10011841 10011841      +       173
## 3 chr21.10011855 chr21 10011855 10011855      +       175
## 4 chr21.10011858 chr21 10011858 10011858      +       175
## 5 chr21.10011861 chr21 10011861 10011861      +       174
## 6 chr21.10011872 chr21 10011872 10011872      +       167
##   numCs1 numTs1 coverage2 numCs2 numTs2 coverage3 numCs3
## 1    173      1        18     18      0        40     34
## 2    164      9        20     19      1        40     18
## 3    175      0        21     21      0        39     29
## 4    131     44        21     20      1        39     31
## 5    147     27        20     15      5        39     13
## 6    160      7        20     19      1        39     34
##   numTs3 coverage4 numCs4 numTs4
## 1      6        14     14      0
## 2     22        14      8      6
## 3     10        14     12      2
## 4      8        13      8      5
## 5     26        13      9      4
## 6      5        14      8      6
```

By default, `unite` function produces bases/regions covered in all samples. That requirement can be relaxed using ”min.per.group” option in `unite` function.

```r
# creates a methylBase object. Only CpGs covered at least
# in 1 sample per group will be returned there were two
# groups defined by the treatment vector given during the
# creation of myobj treatment=c(1,1,0,0)
meth.min <- unite(myobj, min.per.group = 1L)
```

3.2 Sample Correlation

We can check the correlation between samples using `getCorrelation`. This function will either plot scatter plot and correlation coefficients or just print a correlation matrix

```r
getCorrelation(meth, plot = T)

##        test1  test2  ctrl1  ctrl2
## test1 1.0000 0.9253 0.8768 0.8738
## test2 0.9253 1.0000 0.8792 0.8802
## ctrl1 0.8768 0.8792 1.0000 0.9465
## ctrl2 0.8738 0.8802 0.9465 1.0000
```

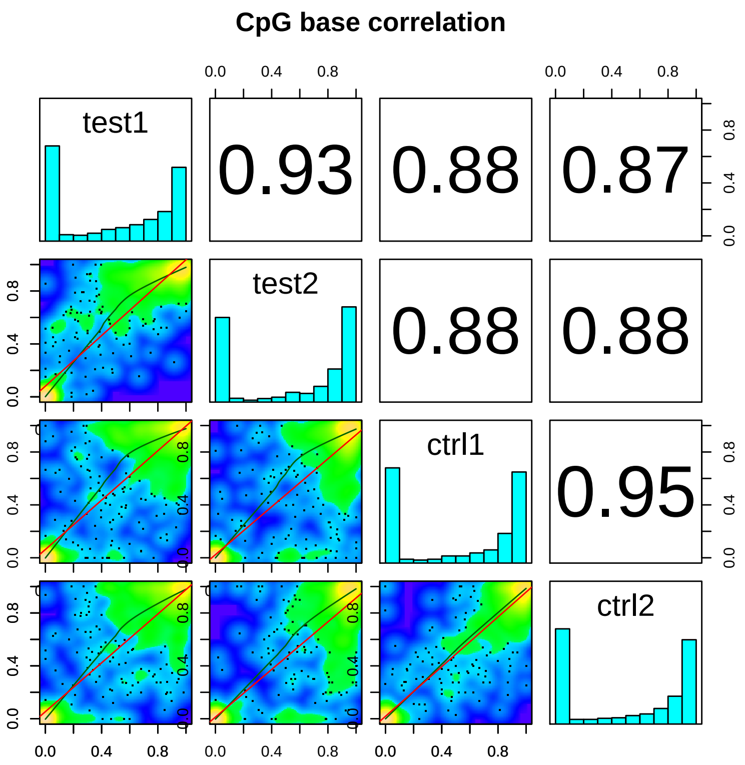

3.3 Clustering samples

We can cluster the samples based on the similarity of their methylation profiles. The following function will cluster the samples and draw a dendrogram.

```
clusterSamples(meth, dist = "correlation", method = "ward",
    plot = TRUE)
```

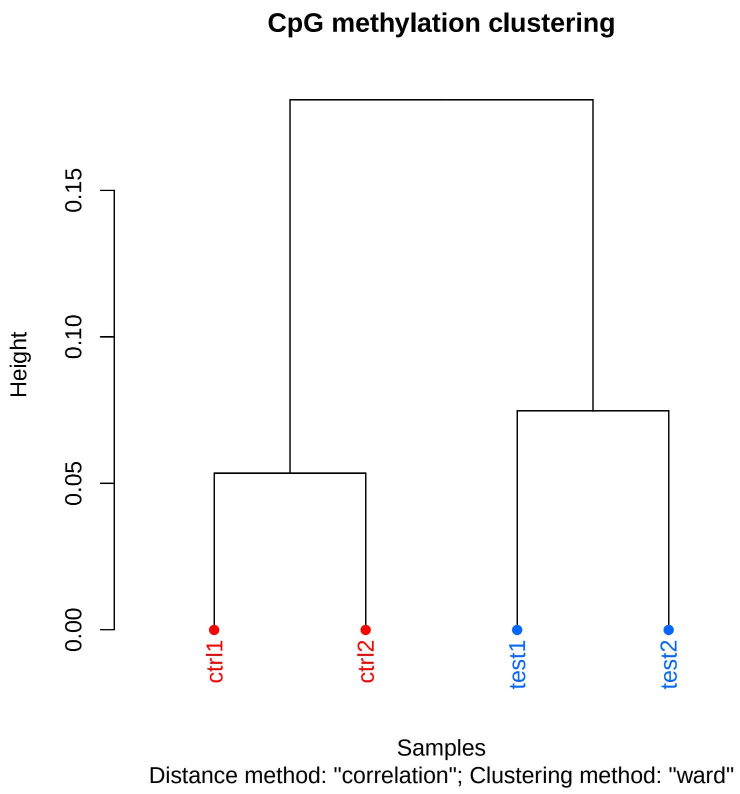


```
##
## Call:
## hclust(d = d, method = HCLUST.METHODS[hclust.method])
##
## Cluster method   : ward
## Distance         : pearson
## Number of objects: 4
##
```

Setting the plot=FALSE will return a dendrogram object which can be manipulated by users or fed in to other user functions that can work with dendrograms.

```
hc <- clusterSamples(meth, dist = "correlation", method = "ward",
    plot = FALSE)
```

We can also do a PCA analysis on our samples. The following function will plot a scree plot for importance of components.

```r
PCASamples(meth, screeplot = TRUE)
```

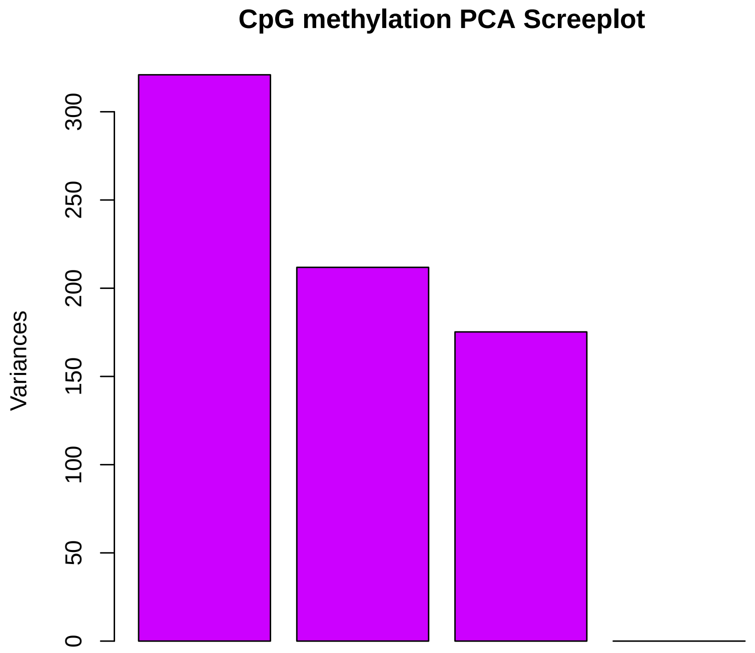

We can also plot PC1 and PC2 axis and a scatter plot of our samples on those axis which will reveal how they cluster.

```r
PCASamples(meth)
```

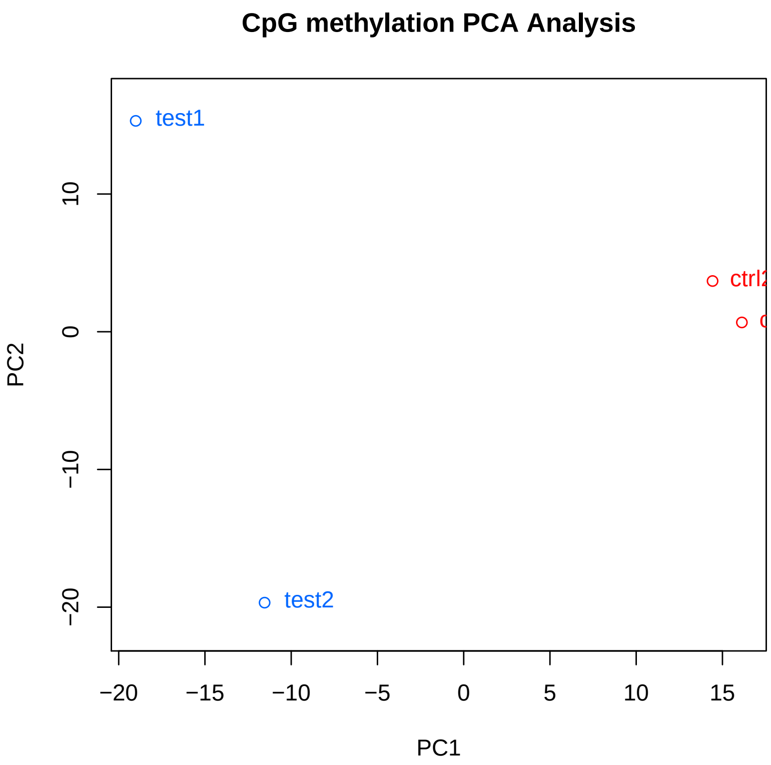

3.4 Tiling windows analysis

For some situations, it might be desirable to summarize methylation information over tiling windows rather than doing base-pair resolution analysis. `methylKit` provides functionality to do such analysis. The function below tiles the genome with windows 1000bp length and 1000bp step-size and summarizes the methylation information on those tiles. In this case, it returns a `methylRawList` object which can be fed into `unite` and `calculateDiffMeth` functions consecutively to get differentially methylated regions.

```
tiles <- tileMethylCounts(myobj, win.size = 1000,
    step.size = 1000)
head(tiles[[1]])

##                        id   chr   start     end strand
## 1 chr21.9764001.9765000 chr21 9764001 9765000      *
## 2 chr21.9820001.9821000 chr21 9820001 9821000      *
## 3 chr21.9837001.9838000 chr21 9837001 9838000      *
## 4 chr21.9849001.9850000 chr21 9849001 9850000      *
```

```
## 5 chr21.9853001.9854000 chr21 9853001 9854000      *
## 6 chr21.9860001.9861000 chr21 9860001 9861000      *
##   coverage numCs numTs
## 1       24     3    21
## 2       13     0    13
## 3       11     0    11
## 4      124    90    34
## 5       34    22    12
## 6       39    38     1
```

3.5 Finding differentially methylated bases or regions

calculateDiffMeth() function is the main function to calculate differential methylation. Depending on the sample size per each set it will either use Fisher's exact or logistic regression to calculate P-values. P-values will be adjusted to Q-values using SLIM method [2].

```
myDiff <- calculateDiffMeth(meth)
```

After q-value calculation, we can select the differentially methylated regions/bases based on q-value and percent methylation difference cutoffs. Following bit selects the bases that have q-value¡0.01 and percent methylation difference larger than 25%. If you specify type="hyper" or type="hypo" options, you will get hyper-methylated or hypo-methylated regions/bases.

```
# get hyper methylated bases
myDiff25p.hyper <- get.methylDiff(myDiff, difference = 25,
    qvalue = 0.01, type = "hyper")
# get hypo methylated bases
myDiff25p.hypo <- get.methylDiff(myDiff, difference = 25,
    qvalue = 0.01, type = "hypo")
#
#
# get all differentially methylated bases
myDiff25p <- get.methylDiff(myDiff, difference = 25,
    qvalue = 0.01)
```

We can also visualize the distribution of hypo/hyper-methylated bases/regions per chromosome using the following function. In this case, the example set includes only one chromosome. The list shows percentages of hypo/hyper methylated bases over all the covered bases in a given chromosome.

```
diffMethPerChr(myDiff, plot = FALSE, qvalue.cutoff = 0.01,
    meth.cutoff = 25)
```

```
## $diffMeth.per.chr
##     chr number.of.hypomethylated
## 1 chr21                       59
##   percentage.of.hypomethylated number.of.hypermethylated
## 1                        6.127                        75
##   percentage.of.hypermethylated
## 1                         7.788
##
## $diffMeth.all
##   percentage.of.hypermethylated number.of.hypermethylated
## 1                         7.788                        75
##   percentage.of.hypomethylated number.of.hypomethylated
## 1                        6.127                       59
##
```

3.5.1 Finding differentially methylated bases using multiple-cores

The differential methylation calculation speed can be increased substantially by utilizing multiple-cores in a machine if available. Both Fisher's Exact test and logistic regression based test are able to use multiple-core option.
The following piece of code will run differential methylation calculation using 2 cores.

```
myDiff <- calculateDiffMeth(meth, num.cores = 2)
```

4 Annotating differentially methylated bases or regions

We can annotate our differentially methylated regions/bases based on gene annotation. In this example, we read the gene annotation from a bed file and annotate our differentially methylated regions with that information. This will tell us what percentage of our differentially methylated regions are on promoters/introns/exons/intergenic region. Similar gene annotation can be fetched using `GenomicFeatures` package available from Bioconductor.org.

```
gene.obj <- read.transcript.features(system.file("extdata",
    "refseq.hg18.bed.txt", package = "methylKit"))
# annotate differentially methylated Cs with
# promoter/exon/intron using annotation data
annotate.WithGenicParts(myDiff25p, gene.obj)

## summary of target set annotation with genic parts
## 133 rows in target set
```

```
## --------------
## --------------
## percentage of target features overlapping with annotation :
##   promoter       exon     intron intergenic
##      27.82      15.04      34.59      57.14
##
##
## percentage of target features overlapping with annotation (with promoter>exon>intron prec
##   promoter       exon     intron intergenic
##      27.82       0.00      15.04      57.14
##
##
## percentage of annotation boundaries with feature overlap :
## promoter     exon   intron
##  0.28604  0.02683  0.17068
##
##
## summary of distances to the nearest TSS :
##    Min. 1st Qu.  Median    Mean 3rd Qu.    Max.
##       5     828   45200   52000   94600  314000
```

Similarly, we can read the CpG island annotation and annotate our differentially methylated bases/regions with them.

```
# read the shores and flanking regions and name the flanks
# as shores and CpG islands as CpGi
cpg.obj <- read.feature.flank(system.file("extdata",
    "cpgi.hg18.bed.txt", package = "methylKit"),
feature.flank.name = c("CpGi",
    "shores"))
#
diffCpGann <- annotate.WithFeature.Flank(myDiff25p,
    cpg.obj$CpGi, cpg.obj$shores, feature.name = "CpGi",
flank.name = "shores")
```

4.1 Regional analysis

We can also summarize methylation information over a set of defined regions such as promoters or CpG islands. The function below summarizes the methylation information over a given set of promoter regions and outputs a `methylRaw` or `methylRawList` object depending on the input.

```
promoters <- regionCounts(myobj, gene.obj$promoters)

head(promoters[[1]])
```

```
##                            id   chr    start      end strand
## 1 chr21.17806094.17808094.NA chr21 17806094 17808094      +
## 2 chr21.10119796.10121796.NA chr21 10119796 10121796      -
## 3 chr21.10011791.10013791.NA chr21 10011791 10013791      -
## 4 chr21.10119808.10121808.NA chr21 10119808 10121808      -
## 5 chr21.15357997.15359997.NA chr21 15357997 15359997      -
## 6 chr21.16023366.16025366.NA chr21 16023366 16025366      +
##   coverage numCs numTs
## 1     1834     7  1827
## 2       79    44    35
## 3     3697  2982   715
## 4       79    44    35
## 5     8613    16  8594
## 6     6296     5  6291
```

4.2 Convenience functions for annotation objects

After getting the annotation of differentially methylated regions, we can get the distance to TSS and nearest gene name using the `getAssociationWithTSS` function.

```
diffAnn <- annotate.WithGenicParts(myDiff25p, gene.obj)

# target.row is the row number in myDiff25p
head(getAssociationWithTSS(diffAnn))

##      target.row dist.to.feature feature.name feature.strand
## 60            1             951    NM_199260              -
## 60.1          2             931    NM_199260              -
## 60.2          3             838    NM_199260              -
## 60.3          4             828    NM_199260              -
## 60.4          5             802    NM_199260              -
## 60.5          6             723    NM_199260              -
```

It is also desirable to get percentage/number of differentially methylated regions that overlap with intron/exon/promoters

```
getTargetAnnotationStats(diffAnn, percentage = TRUE,
    precedence = TRUE)

##   promoter       exon     intron intergenic
##      27.82       0.00      15.04      57.14
```

We can also plot the percentage of differentially methylated bases overlapping with exon/intron/promoters

```r
plotTargetAnnotation(diffAnn, precedence = TRUE, main =
"differential methylation annotation")
```

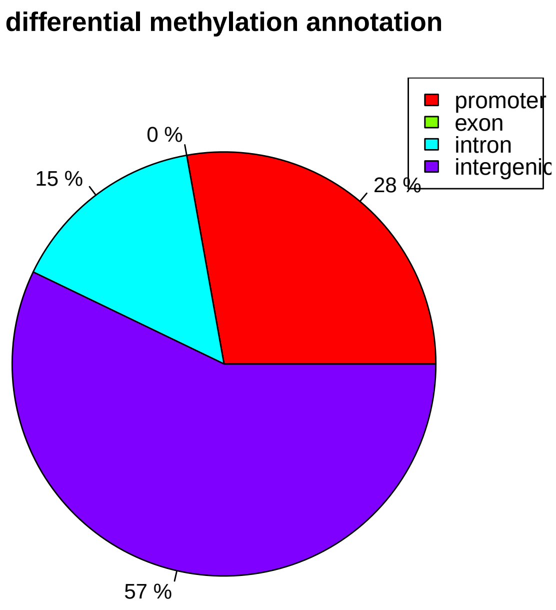

We can also plot the CpG island annotation the same way. The plot below shows what percentage of differentially methylated bases are on CpG islands, CpG island shores and other regions.

```r
plotTargetAnnotation(diffCpGann, col = c("green",
    "gray", "white"), main = "differential methylation
annotation")
```

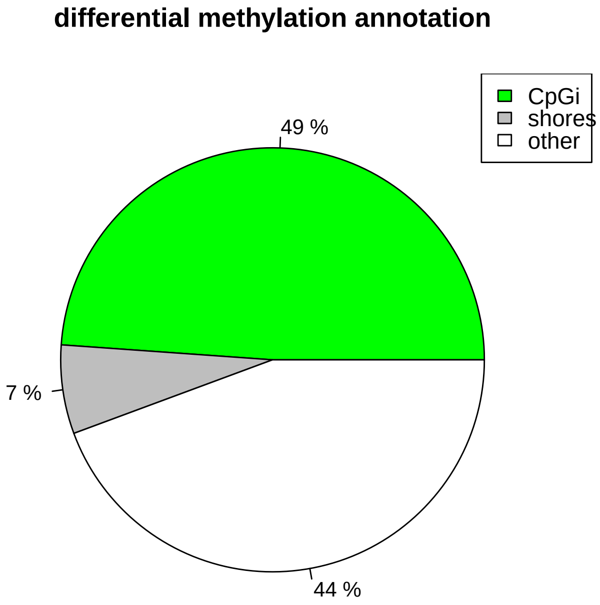

It might be also useful to get percentage of intron/exon/promoters that overlap with differentially methylated bases.

```
getFeatsWithTargetsStats(diffAnn, percentage = TRUE)

## promoter     exon   intron
##  0.28604  0.02683  0.17068
```

5 methylKit convenience functions

5.1 coercion

Most `methylKit` objects (methylRaw,methylBase and methylDiff) can be coerced to `GRanges` objects from `GenomicRanges` package. Coercing methylKit objects to `GRanges` will give users additional flexiblity when customising their analyses.

```
class(meth)

## [1] "methylBase"
## attr(,"package")
## [1] "methylKit"

as(meth, "GRanges")

## GRanges with 963 ranges and 13 elementMetadata cols:
##         seqnames               ranges strand   |
##            <Rle>            <IRanges>  <Rle>   |
##     [1]    chr21 [10011833, 10011833]      +   |
##     [2]    chr21 [10011841, 10011841]      +   |
##     [3]    chr21 [10011855, 10011855]      +   |
##     [4]    chr21 [10011858, 10011858]      +   |
##     [5]    chr21 [10011861, 10011861]      +   |
##     [6]    chr21 [10011872, 10011872]      +   |
##     [7]    chr21 [10011876, 10011876]      +   |
##     [8]    chr21 [10011878, 10011878]      +   |
##     [9]    chr21 [10011925, 10011925]      -   |
##     ...      ...                  ...    ... ...
##   [955]    chr21   [9944505, 9944505]      +   |
##   [956]    chr21   [9944663, 9944663]      -   |
##   [957]    chr21   [9959407, 9959407]      +   |
##   [958]    chr21   [9959541, 9959541]      -   |
##   [959]    chr21   [9959569, 9959569]      -   |
##   [960]    chr21   [9959577, 9959577]      -   |
##   [961]    chr21   [9959644, 9959644]      -   |
##   [962]    chr21   [9959650, 9959650]      -   |
##   [963]    chr21   [9967634, 9967634]      -   |
##                     id coverage1    numCs1    numTs1
##               <factor> <integer> <numeric> <numeric>
##     [1] chr21.10011833       174       173         1
##     [2] chr21.10011841       173       164         9
##     [3] chr21.10011855       175       175         0
##     [4] chr21.10011858       175       131        44
##     [5] chr21.10011861       174       147        27
##     [6] chr21.10011872       167       160         7
##     [7] chr21.10011876       160       148        12
##     [8] chr21.10011878       150       134        16
##     [9] chr21.10011925       120        65        55
##     ...            ...       ...       ...       ...
##   [955]  chr21.9944505        37         2        35
##   [956]  chr21.9944663        61        19        42
##   [957]  chr21.9959407        44        17        27
##   [958]  chr21.9959541        26        12        14
```

```
##   [959]  chr21.9959569        25        17         8
##   [960]  chr21.9959577        25        25         0
##   [961]  chr21.9959644        21         0        21
##   [962]  chr21.9959650        21         6        15
##   [963]  chr21.9967634        10         0        10
##         coverage2    numCs2    numTs2 coverage3    numCs3
##         <integer> <numeric> <numeric> <integer> <numeric>
##     [1]        18        18         0        40        34
##     [2]        20        19         1        40        18
##     [3]        21        21         0        39        29
##     [4]        21        20         1        39        31
##     [5]        20        15         5        39        13
##     [6]        20        19         1        39        34
##     [7]        21        18         3        38        24
##     [8]        20        19         1        37        20
##     [9]        37        21        16        68        21
##     ...       ...       ...       ...       ...       ...
##   [955]       147        56        91        86        79
##   [956]       116        71        45        45        35
##   [957]       118        58        60        52        49
##   [958]        76        44        32        39        37
##   [959]        77        69         8        40        40
##   [960]        77        71         6        40        40
##   [961]        97        50        47        59        52
##   [962]       103        57        46        59        51
##   [963]        61        25        36        93        62
##            numTs3 coverage4    numCs4    numTs4
##         <numeric> <integer> <numeric> <numeric>
##     [1]         6        14        14         0
##     [2]        22        14         8         6
##     [3]        10        14        12         2
##     [4]         8        13         8         5
##     [5]        26        13         9         4
##     [6]         5        14         8         6
##     [7]        14        11         9         2
##     [8]        17        12        12         0
##     [9]        47        20         6        14
##     ...       ...       ...       ...       ...
##   [955]         7        40        25        15
##   [956]        10        31        25         6
##   [957]         3        40        27        13
##   [958]         2        39        32         7
##   [959]         0        39        35         4
##   [960]         0        39        36         3
##   [961]         7        31        14        17
```

```
##   [962]         8        32        21        11
##   [963]        31        56        29        27
##   ---
##   seqlengths:
##    chr21
##       NA
```

```r
class(myDiff)
```

```
## [1] "methylDiff"
## attr(,"package")
## [1] "methylKit"
```

```r
as(myDiff, "GRanges")
```

```
## GRanges with 963 ranges and 3 elementMetadata cols:
##         seqnames               ranges strand   |
##            <Rle>            <IRanges>  <Rle>   |
##     [1]    chr21 [10011833, 10011833]      +   |
##     [2]    chr21 [10011841, 10011841]      +   |
##     [3]    chr21 [10011855, 10011855]      +   |
##     [4]    chr21 [10011858, 10011858]      +   |
##     [5]    chr21 [10011861, 10011861]      +   |
##     [6]    chr21 [10011872, 10011872]      +   |
##     [7]    chr21 [10011876, 10011876]      +   |
##     [8]    chr21 [10011878, 10011878]      +   |
##     [9]    chr21 [10011925, 10011925]      -   |
##     ...      ...                  ...    ... ...
##   [955]    chr21   [9944505, 9944505]      +   |
##   [956]    chr21   [9944663, 9944663]      -   |
##   [957]    chr21   [9959407, 9959407]      +   |
##   [958]    chr21   [9959541, 9959541]      -   |
##   [959]    chr21   [9959569, 9959569]      -   |
##   [960]    chr21   [9959577, 9959577]      -   |
##   [961]    chr21   [9959644, 9959644]      -   |
##   [962]    chr21   [9959650, 9959650]      -   |
##   [963]    chr21   [9967634, 9967634]      -   |
##                        id    qvalue meth.diff
##                  <factor> <numeric> <numeric>
##     [1] chr21.10011833 8.543e-04    10.590
##     [2] chr21.10011841 6.050e-13    46.671
##     [3] chr21.10011855 4.579e-09    22.642
##     [4] chr21.10011858 5.922e-01     2.041
##     [5] chr21.10011861 8.163e-08    41.197
##     [6] chr21.10011872 1.238e-03    16.477
##     [7] chr21.10011876 1.933e-04    24.366
```

```
##     [8] chr21.10011878 3.489e-04    24.694
##     [9] chr21.10011925 8.543e-04    24.095
##     ...            ...       ...       ...
##   [955]  chr21.9944505 0.000e+00   -51.018
##   [956]  chr21.9944663 7.678e-05   -28.100
##   [957]  chr21.9959407 4.839e-08   -36.312
##   [958]  chr21.9959541 3.145e-06   -33.560
##   [959]  chr21.9959569 3.702e-02   -10.623
##   [960]  chr21.9959577 4.923e-01    -2.085
##   [961]  chr21.9959644 3.291e-05   -30.960
##   [962]  chr21.9959650 6.575e-05   -28.314
##   [963]  chr21.9967634 1.028e-03   -25.863
##   ---
##   seqlengths:
##    chr21
##       NA
```

5.2 select

We can also select rows from `methylRaw`, `methylBase` and `methylDiff` objects with "select" function. An appropriate methylKit object will be returned as a result of "select" function.

```
select(meth, 1:10)  # select first 10 rows of a methylBase object

##                 id   chr    start      end strand coverage1
## 1  chr21.10011833 chr21 10011833 10011833      +       174
## 2  chr21.10011841 chr21 10011841 10011841      +       173
## 3  chr21.10011855 chr21 10011855 10011855      +       175
## 4  chr21.10011858 chr21 10011858 10011858      +       175
## 5  chr21.10011861 chr21 10011861 10011861      +       174
## 6  chr21.10011872 chr21 10011872 10011872      +       167
## 7  chr21.10011876 chr21 10011876 10011876      +       160
## 8  chr21.10011878 chr21 10011878 10011878      +       150
## 9  chr21.10011925 chr21 10011925 10011925      -       120
## 10 chr21.10011938 chr21 10011938 10011938      -       134
##    numCs1 numTs1 coverage2 numCs2 numTs2 coverage3 numCs3
## 1     173      1        18     18      0        40     34
## 2     164      9        20     19      1        40     18
## 3     175      0        21     21      0        39     29
## 4     131     44        21     20      1        39     31
## 5     147     27        20     15      5        39     13
## 6     160      7        20     19      1        39     34
## 7     148     12        21     18      3        38     24
## 8     134     16        20     19      1        37     20
```

```
## 9      65     55        37     21     16        68     21
## 10    127      7        36     34      2        74     64
##    numTs3 coverage4 numCs4 numTs4
## 1       6        14     14      0
## 2      22        14      8      6
## 3      10        14     12      2
## 4       8        13      8      5
## 5      26        13      9      4
## 6       5        14      8      6
## 7      14        11      9      2
## 8      17        12     12      0
## 9      47        20      6     14
## 10     10        20     17      3
```

```
select(myDiff, 20:30)  # select rows 10 of a methylDiff object
```

```
##                 id   chr    start      end strand    pvalue
## 20 chr21.10012079 chr21 10012079 10012079      + 1.325e-07
## 21 chr21.10012089 chr21 10012089 10012089      + 6.797e-02
## 22 chr21.10012095 chr21 10012095 10012095      + 9.125e-02
## 23 chr21.10012101 chr21 10012101 10012101      + 8.882e-16
## 24 chr21.10012696 chr21 10012696 10012696      + 2.253e-03
## 25 chr21.10012699 chr21 10012699 10012699      + 1.783e-09
## 26 chr21.10012876 chr21 10012876 10012876      + 4.251e-01
## 27 chr21.10012881 chr21 10012881 10012881      + 1.000e+00
## 28 chr21.10012883 chr21 10012883 10012883      + 4.287e-01
## 29 chr21.10012887 chr21 10012887 10012887      + 1.645e-02
## 30 chr21.10012891 chr21 10012891 10012891      + 8.591e-01
##       qvalue meth.diff
## 20 1.050e-06    26.617
## 21 1.048e-01     9.564
## 22 1.324e-01     5.726
## 23 4.221e-14    39.808
## 24 6.033e-03     9.685
## 25 1.955e-08    44.703
## 26 4.224e-01     3.888
## 27 5.922e-01     0.000
## 28 4.252e-01     3.750
## 29 3.316e-02    20.808
## 30 5.922e-01     0.686
```

5.3 reorganize

`methylBase` and `methylRawList` can be reorganized by `reorganize` function. The function can subset the objects based on provided sample ids, it also cre-

ates a new treatment vector determining which samples belong to which group. Order of sample ids should match the treatment vector order.

```
# creates a new methylRawList object
myobj2 <- reorganize(myobj, sample.ids = c("test1",
    "ctrl2"), treatment = c(1, 0))
# creates a new methylBase object
meth2 <- reorganize(meth, sample.ids = c("test1",
    "ctrl2"), treatment = c(1, 0))
```

5.4 percMethylation

Percent methylation values can be extracted from `methylBase` object by using `percMethylation` function.

```
# creates a matrix containing percent methylation values
perc.meth <- percMethylation(meth)
```

6 Acknowledgements

This package is developed at Weill Cornell Medical College by Altuna Akalin with important code contributions from Sheng Li and Matthias Kormaksson. We wish to thank especially Maria E. Figueroa, Francine Garret-Bakelman, Chrisopher Mason and Ari Melnick for their contribution of ideas and support. Their support and discussions lead to development of methylKit.

7 R session info

```
sessionInfo()

## R version 2.15.0 (2012-03-30)
## Platform: x86_64-apple-darwin9.8.0/x86_64 (64-bit)
##
## locale:
## [1] en_US.UTF-8/en_US.UTF-8/en_US.UTF-8/C/en_US.UTF-8/en_US.UTF-8
##
## attached base packages:
## [1] stats     graphics  grDevices utils     datasets
## [6] methods   base
##
## other attached packages:
## [1] data.table_1.8.0 methylKit_0.5    knitr_0.4
```

```
##
## loaded via a namespace (and not attached):
##  [1] BiocGenerics_0.2.0  codetools_0.2-8
##  [3] digest_0.5.2        evaluate_0.4.2
##  [5] formatR_0.4         GenomicRanges_1.8.3
##  [7] highlight_0.3.1     IRanges_1.14.2
##  [9] KernSmooth_2.23-7   parallel_2.15.0
## [11] parser_0.0-14       plyr_1.7.1
## [13] Rcpp_0.9.10         stats4_2.15.0
## [15] stringr_0.6         tools_2.15.0
```

References

[1] Felix Krueger and Simon R Andrews. Bismark: a flexible aligner and methylation caller for Bisulfite-Seq applications. *Bioinformatics (Oxford, England)*, 27(11):1571–2, June 2011.

[2] Hong-Qiang Wang, Lindsey K Tuominen, and Chung-Jui Tsai. SLIM: a sliding linear model for estimating the proportion of true null hypotheses in datasets with dependence structures. *Bioinformatics (Oxford, England)*, 27(2):225–31, January 2011.
