# Supplementary material for: methylKit: a comprehensive R package for the analysis of genome-wide DNA methylation profiles
Source: Genome Biol. 2012 Oct 3;13(10):R87. doi: 10.1186/gb-2012-13-10-r87 (PMC3491415; doi:10.1186/gb-2012-13-10-r87)
Supplement: Additional file 3 — methylKit documentation. Documentation for functions and classes in the methylKit software package; the most recent software and documentation can be downloaded at [16]. [file gb-2012-13-10-r87-S3.pdf]

Package ‘methylKit’

June 28, 2012

Type Package

Title DNA methylation analysis from high-throughput bisulfite sequencing results

Version 0.5.3

Date 2012-06-16

Author Altuna Akalin, Matthias Kormaksson, Sheng Li

Maintainer Altuna Akalin <aakalin@gmail.com>

Description DNA methylation analysis RRBS,ERRBS,BS-seq

License Artistic-2.0

LazyLoad yes

Depends R (>= 2.15.0), methods

Imports GenomicRanges, IRanges, data.table, parallel

Suggests testthat

Collate
’backbone.R’ ’diffMeth.R’ ’annotate.R’ ’clusterSamples.R’’regionalize.R’ ’read.bismark.R’ ’document_data.R’ ’bedgr

R topics documented:

annotate.WithFeature 2
annotate.WithFeature.Flank 3
annotate.WithGenicParts 4
annotationByFeature-class 4
annotationByGenicParts-class 5
bedgraph 5
calculateDiffMeth 6
clusterSamples 6
convert.bed.df 7
convert.bed2exons 8
convert.bed2introns 8
diffMethPerChr 9
filterByCoverage 9
get.methylDiff 10

getAssembly . . . 11
getAssociationWithTSS . . . 11
getContext . . . 11
getCorrelation . . . 12
getCoverageStats . . . 12
getData . . . 13
getFeatsWithTargetsStats . . . 13
getFlanks . . . 14
getMembers . . . 14
getMethylationStats . . . 15
getTargetAnnotationStats . . . 15
methylBase-class . . . 16
methylBase.obj . . . 16
methylDiff-class . . . 17
methylDiff.obj . . . 17
methylRaw-class . . . 17
methylRawList-class . . . 18
methylRawList.obj . . . 18
normalizeCoverage . . . 18
PCASamples . . . 19
percMethylation . . . 20
plotTargetAnnotation . . . 20
pool . . . 21
read . . . 21
read.bed . . . 22
read.bismark . . . 23
read.feature.flank . . . 24
read.transcript.features . . . 25
regionCounts . . . 25
reorganize . . . 26
select . . . 27
show . . . 27
tileMethylCounts . . . 27
unite . . . 28

Index **29**

`annotate.WithFeature` *function to annotate given GRanges object with a given genomic feature*

Description

function to annotate given GRanges object with a given genomic feature

Usage

```
annotate.WithFeature(target,feature,strand=FALSE,extend=0,feature.name="feat1")
```

Arguments

`target`	a GRanges/or methylDiff object storing chromosome locations to be annotated
`feature`	a GRanges object storing chromosome locations of a feature (can be CpG islands, ChIP-seq peaks, etc)
`strand`	If set to TRUE, annotation features and target features will be overlapped based on strand (def:FALSE)
`extend`	specifiying a positive value will extend the feature on both sides as much as `extend`
`feature.name`	name of the annotation feature. For example: H3K4me1,CpGisland etc.

Value

returns an `annotationByFeature` object

`annotate.WithFeature.Flank` *function to annotate given GRanges object with promoter,exon,intron & intergenic values*

Description

function to annotate given GRanges object with promoter,exon,intron & intergenic values

Usage

```
annotate.WithFeature.Flank(target,feature,flank,feature.name="feat",flank.name="flank",strand=
```

Arguments

`target`	a methylDiff or a granges object storing chromosome locations to be annotated
`feature`	a granges object storing chromosome locations of a feature (can be CpG islands, ChIP-seq peaks, etc)
`flank`	a granges object storing chromosome locations of the flanks of the feature
`feature.name`	string for the name of the feature
`flank.name`	string for the name o
`strand`	If set to TRUE, annotation features and target features will be overlapped based on strand (def:FALSE)

Value

returns an `annotationByFeature` object

Examples

```
data(methylKit)
cpg.obj=read.feature.flank(system.file("extdata", "cpgi.hg18.bed.txt", package = "methylKit"),feature.flank

annotate.WithFeature.Flank(methylDiff.obj,cpg.obj$CpGi,cpg.obj$shores)
```

`annotate.WithGenicParts`
function to annotate given GRanges object with promoter,exon,intron & intergenic values

Description

function to annotate given GRanges object with promoter,exon,intron & intergenic values

Usage

```
annotate.WithGenicParts(target,GRangesList.obj,strand=FALSE)
```

Arguments

`target`	: a methylDiff or a granges object storing chromosome locations to be annotated
`GRangesList.obj`	: A GRangesList object containing GRanges object for promoter,exons,introns and TSSes, or simply output of read.transcript.features function
`strand`	: If set to TRUE, annotation features and target features will be overlapped based on strand (def:FALSE)

Value

`annotationByGenicParts` object

Examples

```
data(methylKit)
gene.obj=read.transcript.features(system.file("extdata", "refseq.hg18.bed.txt", package = "methylKit"))
annotate.WithGenicParts(methylDiff.obj,gene.obj)
```

`annotationByFeature-class`
An S4 class that information on overlap of target features with annotation features

Description

This object is desgined to hold statistics and information about genomic feature overlaps

Slots

`members` a matrix showing overlap of target features with annotation genomic features

`annotation` a named vector of percentages

`precedence` a named vector of percentages

`num.hierarchica` vector

`no.of.OlapFeat` vector

`perc.of.OlapFeat` vector

`annotationByGenicParts-class` *An S4 class that information on overlap of target features with annotation features*

Description

This object is desgined to hold statistics and information about genomic feature overlaps

Slots

`members` a matrix showing overlap of target features with annotation genomic features

`annotation` a named vector of percentages

`precedence` a named vector of percentages

`num.hierarchica` vector

`no.of.OlapFeat` vector

`perc.of.OlapFeat` vector

dist.to.TSS a data frame showing distances to TSS and gene/TSS names and strand

---

`bedgraph` *GETs bedgraph from methylRaw, methylRawList and methylDiff objects*

Description

Convert `methylRaw`, `methylRawList` or `methylDiff` object into a bedgraph format

Usage

```
bedgraph(methylObj,file.name=NULL,col.name,unmeth=FALSE,log.transform=FALSE,negative=FALSE,add
```

Arguments

`methylObj`	a `methylRaw` or `methlRawList` object
`file.name`	Default: NULL, if given a bedgraph file will be written, if NULL a data.frame or a list of data frames will be returned
`col.name`	name of the column in `methylRaw`, `methylRawList` or `methylDiff` objects to be used as a score for the bedgraph. For `methylDiff`, col.name must be one of the following 'pvalue','qvalue', 'meth.diff'. For `methylRaw` and `methylRawList` it must be one of the following 'coverage', 'numCs','numTs', 'perc.meth'
`unmeth`	when working with `methylRaw` or `methylRawList` objects should you output unmethylated C percentage this makes it easier to see the unmethylated bases because their will be zero. Only invoked when file.name is not NULL.
`log.transform`	Default FALSE, If TRUE the score column of the bedgraph wil be in log10 scale. Ignored when col.name='perc.meth'
`negative`	Default FALSE, If TRUE, the score column of the bedgraph will be multiplied by -1. Ignored when col.name='perc.meth'
`add.on`	additional string to be add on the track line of bedgraph. can be viewlimits,priority etc. Check bedgraph track line options at UCSC browser

Value

RETURNS a data.frame or list of data.frames if file.name=NULL, if a file.name given appropriate bed file will be written to that file

---

`calculateDiffMeth` *Calculates differential methylation statistics*

Description

Calculates differential methylation statistics

Usage

```
calculateDiffMeth(.Object,slim=TRUE,weigthed.mean=TRUE,num.cores=1)
```

Arguments

`.Object`	a methylBase object to calculate differential methylation
`slim`	If TRUE(default) SLIM method will be used for P-value adjustment.If FALSE, p.adjust with method="BH" option will be used for P-value correction.
`weigthed.mean`	calculate the mean methylation difference between groups using read coverage as weights
`num.cores`	integer for denoting how many cores should be used for differential methylation calculations (only can be used in machines with multiple cores)

Value

a methylDiff object containing the differential methylation statistics and locations

Note

The function either uses a logistic regression (when there are multiple samples per group) or fisher's exact when there is one sample per group.

`clusterSamples` *CpG Dinucleotide Methylation Hierarchical Cluster Analysis*

Description

CpG Dinucleotide Methylation Hierarchical Cluster Analysis

Usage

```
clusterSamples(.Object, dist="correlation",
method="ward", plot=TRUE)
```

Arguments

`.Object`	a `methylBase` object
`dist`	the distance measure to be used. This must be one of "`correlation`", "`euclidean`", "`maximum`", "`manhattan`", "`canberra`", "`binary`" or "`minkowski`". Any unambiguous substring can be given. (default:"`correlation`")
`method`	the agglomeration method to be used. This should be (an unambiguous abbreviation of) one of "`ward`", "`single`", "`complete`", "`average`", "`mcquitty`", "`median`" or "`centroid`". (default:"`ward`")
`plot`	a logical value indicating whether to plot hierarchical clustering. (default:TRUE)

Value

a `tree` object of a hierarchical cluster analysis using a set of dissimilarities for the n objects being clustered.

`convert.bed.df` *convert a data frame read-in from a bed file to a GRanges object*

Description

convert a data frame read-in from a bed file to a GRanges object

Usage

```
convert.bed.df(bed)
```

Arguments

`bed`	a data.frame where column order and content resembles a bed file with 12 columns

Value

`GRanges` object

Note

one bed track per file is only accepted, the bed files with multiple tracks will cause en error

---

`convert.bed2exons` *convert a data frame read-in from a bed file to a GRanges object for exons*

Description

convert a data frame read-in from a bed file to a GRanges object for exons

Usage

```
convert.bed2exons(bed.df)
```

Arguments

`bed.df`	a data.frame where column order and content resembles a bed file with 12 columns

Value

`GRanges` object

Note

one bed track per file is only accepted, the bed files with multiple tracks will cause en error

---

`convert.bed2introns` *convert a data frame read-in from a bed file to a GRanges object for introns*

Description

convert a data frame read-in from a bed file to a GRanges object for introns

Usage

```
convert.bed2introns(bed.df)
```

Arguments

`bed.df`	a data.frame where column order and content resembles a bed file with 12 columns

Value

`GRanges` object

Note

one bed track per file is only accepted, the bed files with multiple tracks will cause en error

---

`diffMethPerChr` *Gets and plots the number of hyper/hypo methylated regions per chromosome*

Description

This accessor function gets the nearest TSS, its distance to target feature,strand and name of TSS/gene from annotationByGenicParts object.

Usage

```
diffMethPerChr(x,plot=T,qvalue.cutoff=0.01,
meth.cutoff=25,exclude=NULL,...)
```

Arguments

`x`	a `annotationByFeature` object
`plot`	TRUE|FALSE. If TRUE horizontal barplots for proportion of hypo/hyper methylated bases/regions
`qvalue.cutoff`	cutoff for q-value
`meth.cutoff`	cutoff for percent methylation difference
`exclude`	names of chromosomes to be excluded
`...`	extra graphical parameters to be passed to `barplot` function

Value

plots a piechart or a barplot for percentage of the target features overlapping with annotation

---

`filterByCoverage` *filter methylRaw and methylRawList object based on read coverage*

Description

This function filters `methylRaw` and `methylRawList` objects. You can filter based on lower read cutoff or high read cutoff. Higher read cutoff is usefull to eliminate PCR effects Lower read cutoff is usefull for doing better statistical tests.

Usage

```
filterByCoverage(methylObj,lo.count=NULL,lo.perc=NULL,hi.count=NULL,hi.perc=NULL)
```

Arguments

`methylObj`	a `methylRaw` or `methylRawList` object
`lo.count`	An integer for read counts.Bases/regions having lower coverage than this count is discarded
`lo.perc`	A double [0-100] for percentile of read counts. Bases/regions having lower coverage than this percentile is discarded
`hi.count`	An integer for read counts. Bases/regions having higher coverage than this is count discarded
`hi.perc`	A double [0-100] for percentile of read counts. Bases/regions having higher coverage than this percentile is discarded

Value

`methylRaw` or `methylRawList` object depending on input object

`get.methylDiff` *gets differentially methylated regions/bases based on cutoffs*

Description

gets differentially methylated regions/bases based on cutoffs

Usage

```
get.methylDiff(.Object,difference=25,qvalue=0.01,type="all")
```

Arguments

`.Object`	a methylDiff object
`difference`	cutoff for absolute value of methylation change between test and control (default:25)
`qvalue`	cutoff for qvalue of differential methylation statistic (default:0.01)
`type`	one of the "hyper","hypo" or "all" strings. Specifies what type of differentially menthylated bases/regions should be returned. For retrieving Hyper-methylated regions/bases type="hyper", for hypo-methylated type="hypo" (default:"all")

Value

a methylDiff object containing the differential methylated locations satisfying the criteria

getAssembly — *get assembly of the genome*

Description

get assembly of the genome

Arguments

`x`	a methylBase object

Value

the assembly string for the object

getAssociationWithTSS — *Get distance to nearest TSS and gene id from annotationByGenicParts*

Description

This accessor function gets the nearest TSS, its distance to target feature,strand and name of TSS/gene from annotationByGenicParts object

Usage

```
getAssociationWithTSS(x)
```

Arguments

`x`	a `annotationByGenicParts` object

Value

RETURNS a data.frame containing row number of the target features,distance of target to nearest TSS, TSS/Gene name, TSS strand

getContext — *get the context of methylation*

Description

get the context of methylation

Arguments

`x`	a methylBase/methylRaw/methylDiff object

Value

the context of methylation string

`getCorrelation` *get correlation between samples in methylBase object*

Description

get correlation between samples in methylBase object

Usage

```
getCorrelation(.Object,method="pearson",plot=FALSE)
```

Arguments

`.Object`	a methylBase object
`method`	a character string indicating which correlation coefficient (or covariance) is to be computed (default:"pearson", other options are "kendall" and "spearman")
`plot`	scatterPlot if TRUE (default:FALSE)

Value

a correlation matrix object and plot scatterPlot

`getCoverageStats` *get coverage stats from methylRaw object*

Description

get coverage stats from methylRaw object

Usage

```
getCoverageStats(.Object,plot=FALSE,both.strands=FALSE,labels=TRUE,...)
```

Arguments

`.Object`	a `methylRaw` object
`plot`	plot a histogram of coverage if TRUE (default:FALSE)
`both.strands`	do stats and plot for both strands if TRUE (default:FALSE)
`labels`	should the bars of the histrogram have labels showing the percentage of values in each bin (default:TRUE)
`...`	options to be passed to `hist` function

Value

a summary of coverage statistics or plot a histogram of coverage

getData — *gets the data slot from the methylBase object*

Description

The data retrived from this function is of a `data.frame`. This is basically containing all relevant methylation information per region

Arguments

`x`	a methylBase object

Value

data.frame for methylation events

getFeatsWithTargetsStats — *Get the percentage/count of annotation features overlapping with target features from annotationByFeature*

Description

This function retrieves percentage/number of annotation features overlapping with targets. For example, if `annotationByFeature` object is containing statistics of differentially methylated regions overlapping with gene annotation. This function will return number/percentage of introns,exons and promoters overlapping with differentially methylated regions.

Usage

```
getFeatsWithTargetsStats(x,percentage=TRUE)
```

Arguments

`x`	a `annotationByFeature` object
`percentage`	TRUE|FALSE. If TRUE percentage of annotation features will be returned. If FALSE, number of annotation features will be returned

Value

RETURNS a vector of percentages or counts showing quantity of annotation features overlapping with target features

getFlanks	*a function to get upstream and downstream adjecent regions to a genomic feature such as CpG islands*

Description

a function to get upstream and downstream adjecent regions to a genomic feature such as CpG islands

Usage

```
getFlanks(grange,flank=2000,clean=T)
```

Arguments

`grange`	GRanges object for the feature
`flank`	number of basepairs for the flanking regions
`clean`	If set to TRUE, flanks overlapping with other main features will be trimmed, and overlapping flanks will be removed this will remove multiple counts when other features overlap with flanks

Value

GRanges object for flanking regions

getMembers	*Get the membership slot of annotationByFeature*

Description

Membership slot defines the overlap of target features with annotation features For example, if a target feature overlaps with an exon

Usage

```
getMembers(x)
```

Arguments

`x`	a `annotationByFeature` object

Value

RETURNS a matrix showing overlap of target features with annotation features. 1 for overlap, 0 for non-overlap

`getMethylationStats` *get Methylation stats from methylRaw object*

Description

get Methylation stats from methylRaw object

Usage

```
getMethylationStats(.Object,plot=FALSE,both.strands=FALSE,labels=TRUE,...)
```

Arguments

`.Object`	a `methylRaw` object
`plot`	plot a histogram of Methylation if TRUE (deafult:FALSE)
`both.strands`	do plots and stats for both strands seperately if TRUE (deafult:FALSE)
`labels`	should the bars of the histrogram have labels showing the percentage of values in each bin (default:TRUE)
`...`	options to be passed to `hist` function.

Value

a summary of Methylation statistics or plot a histogram of coverage

`getTargetAnnotationStats` *Get the percentage of target features overlapping with annotation from annotationByFeature*

Description

This function retrieves percentage/number of target features overlapping with annotation

Usage

```
getTargetAnnotationStats(x,percentage=TRUE,precedence=TRUE)
```

Arguments

`x`	a `annotationByFeature` object
`percentage`	TRUE|FALSE. If TRUE percentage of target features will be returned. If FALSE, number of target features will be returned
`precedence`	TRUE|FALSE. If TRUE there will be a hierachy of annotation features when calculating numbers (with promoter>exon>intron precedence) That means if a feature overlaps with a promoter it will be counted as promoter overlapping only, or if it is overlapping with a an exon but not a promoter, it will be counted as exon overlapping only whether or not it overlaps with an intron.

Value

RETURNS a vector of percentages or counts showing quantity of target features overlapping with annotation

---

`methylBase-class` *An S4 class that holds base-pair resolution methylation information for multiple experiments, only bases that are covered in all experiments are held in this class*

Description

extends data.frame and creates an object that holds methylation information and genomic location

Slots

`sample.ids`: character vector for ids of samples in the object

`assembly`: name of the genome assembly

`context`: context of methylation. Ex: CpG,CpH,CHH, etc

`treatment`: treatment vector denoting which samples are test and control

`coverage.index`: vector denoting which columns in the data correspons to coverage values

`numCs.index`: vector denoting which columns in the data correspons to number of methylatedCs values

`numTs.index`: vector denoting which columns in the data correspons to number of unmethylated Cs values

`resolution`: resolution of methylation information, allowed values: 'base' or 'region'

`methylBase.obj` *Example methylBase object.*

Description

methylKit has several objects. This data set includes examples of the following objects: `methylBase`, `methylDiff` and `methylRawList`. You can load the data using `data(methylKit)`

Format

methylBase.obj object stores the location and methylation information for bases that are covered in all samples. `methylBase` partially extends `data.frame` S3 class.

methylDiff-class *An S4 class that holds differential methylation information*

Description

This object is desgined to hold statistics and locations for differentially methylated regions/bases

Slots

`sample.ids` ids/names of samples in a vector

`assembly` a name of genome assembly, such as :hg18,mm9, etc

`context` numeric vector identifying which samples are which group

`treatment` numeric vector identifying which samples are which group

`destranded` logical denoting if methylation inormation is destranded or not

`resolution` string either 'base' or 'region' defining the resolution of methylation information

`.Data` data.frame holding the locations and statistics

methylDiff.obj *Example methylKit objects.*

Description

methylKit has several objects. This data set includes examples of the following objects: `methylBase`, `methylDiff` and `methylRawList`. You can load the data using `data(methylKit)`

Format

The Differential methylation information is stored in methylDiff.obj object. `methylBase` partially extends `data.frame` S3 class.

methylRaw-class *An S4 class for holding raw methylation data from alignment pipeline.*

Description

This object stores the raw mehylation data that is read in through read function and extends data.frame

Slots

`sample.id`: string for an identifier of the sample

`assembly`: string for genome assembly, ex: hg18,hg19,mm9

`context`: methylation context string, ex: CpG,CpH,CHH, etc.

`resolution`: resolution of methylation information, 'base' or 'region'

methylRawList-class *An S4 class for holding a list of methylRaw objects.*

Description

This object stores the list of raw mehylation data that is read in through read function and extends data.frame

Slots

`treatment`: numeric vector denoting control and test samples

methylRawList.obj *Example methylRawList object.*

Description

methylKit has several objects. This data set includes examples of the following objects: `methylBase`, `methylDiff` and `methylRawList`. You can load the data using `data(methylKit)`

Format

Methylation data from multiple the samples regardless of common coverage are stored in methylRawList.obj object. `methylRawList` extends `list` S3 class

normalizeCoverage *normalize read coverage between samples*

Description

The function normalizes coverage values between samples using a scaling factor derived from differences between mean or median of coverage distributions

Usage

```
normalizeCoverage(obj,method="median")
```

Arguments

`obj`	`methylRawList` object
`method`	a string "mean" or "median" which denotes median or mean should be used to calculate scaling factor. (Default:median)

Value

a `methylRawList` object

Author(s)

Altuna Akalin

Examples

```
library(methylKit)
data(methylKit)
newObj=normalizeCoverage(methylRawList.obj)
```

`PCASamples` *CpG Dinucleotide Methylation Principal Components Analysis*

Description

CpG Dinucleotide Methylation Principal Components Analysis

Usage

```
PCASamples(.Object, cor=TRUE, screeplot=FALSE,
adj.lim=c(0.0004,0.1),scale=TRUE,center=TRUE,comp=c(1,2),transpose=TRUE,sd.threshold=0,obj.ret
```

Arguments

`.Object`	a `methylBase` object
`cor`	[Not used anymore] cor a logical value indicating whether the calculation should use the correlation matrix or the covariance matrix. (default: TRUE)
`screeplot`	a logical value indicating whether to plot the variances against the number of the principal component. (default: FALSE)
`adj.lim`	a vector indicating the propotional adjustment of xlim (adj.lim[1]) and ylim (adj.lim[2]). (default: c(0.0004,0.1))
`scale`	logical indicating if `prcomp` should scale the data to have unit variance or not (default: TRUE)
`center`	logical indicating if `prcomp` should center the data or not (default: TRUE)
`comp`	vector of integers with 2 elements specifying which components to be plotted.
`transpose`	if TRUE (default) percent methylation matrix will be transposed, this is equivalent to doing PCA on variables that are regions/bases. The resulting plot will location of samples in the new coordinate system if FALSE the variables for the matrix will be samples and the resulting plot whill show how each sample (variable) contributes to the principle component. the samples that are highly correlated should have similar contributions to the principal components.
`sd.threshold`	standard deviation threshold to remove bases/regions that have dev. lower than this threshold. if NULL no standard deviation will be calculated and this threshold will not be applied.
`obj.return`	if the result of `prcomp` function should be returned or not. Default:FALSE

Value

The form of the value returned by `PCASamples` is the summary of principal component analysis by `prcomp`.

Note

cor option is not in use anymore, since `prcomp` is used for PCA analysis instead of `princomp`

`percMethylation` *get percent methylation scores from methylBase object*

Description

get percent methylation scores from methylBase object

Usage

```
percMethylation(methylBase.obj)
```

Arguments

`methylBase.obj`	a methylBase object

Value

matrix with percent methylation values per base/region across all samples, row names would be base/region identifiers

---

`plotTargetAnnotation` *Plot annotation categories from annotationByGenicParts or annotationByFeature*

Description

This function plots a pie or bar chart for showing percentages of targets annotated by genic parts or other query features

Arguments

`x`	a `annotationByFeature` or `annotationByGenicParts` object
`precedence`	TRUE|FALSE. If TRUE there will be a hierachy of annotation features when calculating numbers (with promoter>exon>intron precedence). This option is only valid when `x` is a `annotationByGenicParts` object
`col`	a vector of colors for piechart or the bar plot
`...`	graphical parameters to be passed to `pie` or `barplot` functions<br>usage plotTargetAnnotation(x,precedence=TRUE,col,...)

Value

plots a piechart or a barplot for percentage of the target features overlapping with annotation

`pool` *function pools replicates within groups to a single sample per group*

Description

The function sums up coverage, numCs and numTs values within each group so one representative sample for each group will be created in a new methylBase object

Usage

```
pool(obj,sample.ids)
```

Arguments

`obj`	`methylBase` object with two groups or more and each group should have multiple samples
`sample.ids`	a character vector of new sample.ids ex:c("test","control"), should follow the same order as unique treatment vector, and should be equal to the length of the unique treatment vector

Value

a `methylBase` object

Author(s)

Altuna Akalin

Examples

```
library(methylKit)
data(methylKit)
newBase=pool(methylBase.obj,sample.ids=c("test","control"))
```

`read` *read file(s) to a methylrawList or methylraw object*

Description

read a list of locations or one location and create a methylrawList or methylraw object

Usage

```
read(location,sample.id,assembly,pipeline="amp",header=T,
context="CpG",resolution="base",treatment)
```

Arguments

location	file location(s), either a list of locations (each a character string) or one location string
sample.id	sample.id(s)
assembly	a string that defines the genome assembly such as hg18, mm9
header	if the input file has a header or not (default: TRUE)
pipeline	name of the alignment pipeline, currently only supports amp or bismark (default: 'amp')
resolution	designates whether methylation information is base-pair resolution or regional resolution. allowed values 'base' or 'region'. Default 'base'
treatment	a vector contatining 0 and 1 denoting which samples are control which samples are test
context	methylation context string, ex: CpG,CpH,CHH, etc. (default:CpG)

Value

returns methylRaw or methylRawList

---

read.bed *read a bed file and convert it to GRanges*

Description

read a bed file and convert it to GRanges

Usage

```
read.bed(location,remove.unsual=TRUE)
```

Arguments

location	location of the file, a character string such as: "/home/user/my.bed"
remove.unsual	if TRUE(default) remove the chromomesomes with unsual names, mainly random chromsomes etc

Value

GRanges object

Note

one bed track per file is only accepted, the bed files with multiple tracks will cause en error

read.bismark — *Function to read in pecent methylation scores from sorted Bismark SAM files*

Description

The function calls methylation percentage per base from sorted Bismark SAM files. Bismark is a popular aligner for high-throughput bisulfite sequencing experiments and it outputs its results in SAM format by default. Bismark SAM format contains aligner specific tags which are absolutely necessary for methylation percentage calling. SAM files from other aligners will not work with this function.

Usage

```
read.bismark(location,sample.id,assembly,save.folder=NULL,save.context=c("CpG"),read.context="
```

Arguments

location	location of sam file(s). If multiple files are given this arugment must be a list.
sample.id	the id(s) of samples in the same order as file. If multiple sam files are given this arugment must be a list.
save.folder	The folder which will be used to save methylation call files, if set to NULL no methylation call file will be saved as a text file. The files saved can be read into R in less time using `read` function in `methylKit`
save.context	A character vector consisting following strings: "CpG","CHG","CHH". The methylation percentages for these methylation contexts will be saved to save.folder
read.context	One of the 'CpG','CHG','CHH' or 'none' strings. Determines what type of methylation context will be read-in to the memory which can be immediately used for analysis. If given as 'none', read.bismark will not return any object, but if a save.folder argument given it will save the methylation percentage call files.
assembly	string that determines the genome assembly. Ex: mm9,hg18 etc.
nolap	if set to TRUE and the SAM file has paired-end reads, the one read of the overlapping paired-end read pair will be ignored for methylation calling.
mincov	minimum read coverage to call a methylation status for a base.
minqual	minimum phred quality score to call a methylation status for a base.
phred64	logical (default: FALSE) you will not need to set this TRUE, Currently bismark gives only phred33 scale
treatment	treatment vector only to be used when location and sample.id parameters are `lists` and you are trying to read-in multiple samples that are related to eachother in down-stream analysis.

Value

`methylRaw` or `methylRawList` object

Examples

```
# read.bismark("/Users/altuna/Dropbox\\ Encore/Dropbox/temp/data/bismark_6.4_trial/test.fastq_bismark.sam",
#    save.folder="/Users/altuna",save.context="CpG",read.context="none")
# file.list2=list(system.file("extdata", "test.fastq_bismark.sorted.min.sam", package = "methylKit"),
#               system.file("extdata", "test.fastq_bismark.sorted.min.sam", package = "methylKit"),
#               system.file("extdata", "test.fastq_bismark.sorted.min.sam", package = "methylKit"),
#               system.file("extdata", "test.fastq_bismark.sorted.min.sam", package = "methylKit"))
#
# objs=read.bismark(location=file.list2
#             ,sample.id=list("test1","test2","ctrl1","ctrl1"),assembl="hg18",save.folder=NULL,save.context
#                                    nolap=FALSE,mincov=10,minqual=20,phred64=FALSE,treatment=c(1,1,0,0))
```

`read.feature.flank` *a function to read-in genomic features and their upstream and downstream adjecent regions such as CpG islands and their shores*

Description

a function to read-in genomic features and their upstream and downstream adjecent regions such as CpG islands and their shores

Usage

```
read.feature.flank(location,remove.unsual=TRUE,flank=2000,clean=TRUE,feature.flank.name=NULL)
```

Arguments

`location`	for the bed file of the feature
`flank`	number of basepairs for the flanking regions
`clean`	If set to TRUE, flanks overlapping with other main features will be trimmed
`remove.unsual`	remove chromsomes with unsual names random, Un and antyhing with "_" character
`feature.flank.name`	the names for feature and flank ranges, it should be a character vector of length 2. example: c("CpGi","shores")

Value

a GenomicRangesList contatining one GRanges object for flanks and one for GRanges object for the main feature.

Examples

```
# location of the example CpG file
my.loc=system.file("extdata", "cpgi.hg18.bed.txt", package = "methylKit")
cpg.obj=read.feature.flank(location=my.loc,feature.flank.name=c("CpGi","shores"))
```

read.transcript.features *function reading exon intron, promoter structure from a given bed file*

Description

function reading exon intron, promoter structure from a given bed file

Usage

```
read.transcript.features(location,remove.unusual=TRUE,up.flank=1000,down.flank=1000,unique.prom
```

Arguments

location	location of the bed file with 12 or more columns
remove.unusual	remove the chromomesomes with unusual names, mainly random chromsomes etc
up.flank	up-stream from TSS to detect promoter boundaries
down.flank	down-stream from TSS to detect promoter boundaries
unique.prom	get only the unique promoters, promoter boundaries will not have a gene name if you set this option to be TRUE

Value

a `GRangesList` containing locations of exon/intron/promoter/TSS

Note

one bed track per file is only accepted, the bed files with multiple tracks will cause en error

regionCounts *GETs regional counts for given GRanges or GRangesList object*

Description

Convert `methylRaw` or `methylRawList` object into regional counts for a given GRanges or GRangesList object.

Usage

```
regionCounts(methylObj,regions,cov.bases=0,strand.aware=FALSE)
```

Arguments

methylObj	a `methylRaw` or `methlRawList` object
regions	a GRanges or GRangesList object.
cov.bases	number minimum bases covered per region (Default:0). Only regions with base coverage above this threshold are returned.
strand.aware	if set to TRUE only CpGs that match the strand of the region will be summarized

Value

RETURNS a new methylRaw or methylRawList object

`reorganize` *reorganize methylRawList and methylBase objects by creating new objects from subset of samples*

Description

Create a new `methylRawList` or `methylBase` object by selecting a subset of samples from the input object, which is a `methylRawList` or `methylBase` object. You can use the function to partition a large methylRawList or methylBase object to smaller object based on sample ids or when you want to reorder samples and treatmet vector.

Usage

```
reorganize(methylObj,sample.ids,treatment)
```

Arguments

`methylObj`	a `methylRawList` or `methylBase` object
`sample.ids`	a vector for sample.ids to be subset. Order is important and the order should be similar to treatment. sample.ids should be a subset or reordered version of sample ids in the input object.
`treatment`	treatment vector, should be same length as sample.ids vector

Value

RETURNS a `methylRawList` or `methylBase` object depending on the input object

Examples

```
# this is a list of example files, ships with the package
file.list=list( system.file("extdata", "test1.myCpG.txt", package = "methylKit"),
system.file("extdata", "test2.myCpG.txt", package = "methylKit"),
system.file("extdata", "control1.myCpG.txt", package = "methylKit"),
system.file("extdata", "control2.myCpG.txt", package = "methylKit") )


# read the files to a methylRawList object: myobj
myobj=read( file.list,
sample.id=list("test1","test2","ctrl1","ctrl2"),assembly="hg18",pipeline="amp",treatment=c(1,1,0,0))
meth=unite(myobj,destrand=TRUE)

myobj2=reorganize(myobj,sample.ids=c("test1","ctrl2"),treatment=c(1,0) )
meth2 =reorganize(meth,sample.ids=c("test1","ctrl2"),treatment=c(1,0) )
```

`select` *selects rows from of methylRaw.methylBase and methylDiff objects*

Description

selects rows from of methylRaw.methylBase and methylDiff objects

Examples

```
# select(methylRaw.obj,1:100) # selects first hundred rows, returns a methylRaw object
# select(methylBase.obj,1:100)
# select(methylDiff.obj,1:100)
```

`show` *show method for some of the methylKit classes*

Description

show method for some of the methylKit classes

`tileMethylCounts` *Get methylated/unmethylated base counts for tilling windows*

Description

The function summarizes methylated/unmethylated base counts over tilling windows accross genome. This function can be used when differential methylated analysis is preferable to tilling windows instead of base pairs.

Usage

```
tileMethylCounts(methylObj,win.size=1000,step.size=1000,cov.bases=0)
```

Arguments

`methylObj`	`methylRaw` or `methylRawList` object containing base pair resolution methylation information
`win.size`	an integer for the size of the tiling windows
`step.size`	an integer for the step size of tiling windows
`cov.bases`	minimum number of bases to be covered in a given window

Value

`methylRaw` or `methylRawList` object

`unite` *unites methylRawList to a single table*

Description

This functions unites `methylRawList` object that only bases with coverage from all samples are retained. The resulting object is a class of `methylBase`

Usage

```
unite(.Object,destrand=FALSE,min.per.group=NULL)
```

Arguments

`.Object`	a methylRawList object to be merged by common locations covered by reads
`destrand`	if TRUE, reads covering both strands of a CpG dinucleotide will be merged, do not set to TRUE if not only interested in CpGs (default: FALSE). If the methylRawList object contains regions rather than bases setting destrand to TRUE will have no effect.
`min.per.group`	an integer denoting minimum number of samples per replicate needed to cover a region/base. By default only regions/bases that are covered in all samples are united as methylBase object, however by supplying an integer for this argument users can control how many samples needed to cover region/base to be united as methylBase object. For example, if min.per.group set to 2 and there are 3 replicates per condition, the bases/regions that are covered in at least 2 replicates will be united and missing data for uncovered bases/regions will appear as NAs.

Value

a methylBase object

Examples

```
## myobj is a methylRawList object
# unite(myobj)
# unite(myobj,min.per.group=1L) # at least 1 sample per group should be covered for any given base/region
# unite(myobj,destrand=TRUE)
```

Index

annotate.WithFeature, 2
annotate.WithFeature,GRanges,GRanges-method (annotate.WithFeature), 2
annotate.WithFeature,methylDiff,GRanges-method (annotate.WithFeature), 2
annotate.WithFeature.Flank, 3
annotate.WithFeature.Flank,GRanges,GRanges,GRanges-method (annotate.WithFeature.Flank), 3
annotate.WithFeature.Flank,methylDiff,GRanges,GRanges-method (annotate.WithFeature.Flank), 3
annotate.WithGenicParts, 4
annotate.WithGenicParts,GRanges,GRangesList-method (annotate.WithGenicParts), 4
annotate.WithGenicParts,methylDiff,GRangesList-method (annotate.WithGenicParts), 4
annotationByFeature-class, 4
annotationByGenicParts-class, 5

bedgraph, 5
bedgraph,methylDiff-method (bedgraph), 5
bedgraph,methylRaw-method (bedgraph), 5
bedgraph,methylRawList-method (bedgraph), 5

calculateDiffMeth, 6
calculateDiffMeth,methylBase-method (calculateDiffMeth), 6
clusterSamples, 6
clusterSamples,methylBase-method (clusterSamples), 6
convert.bed.df, 7
convert.bed.df,data.frame-method (convert.bed.df), 7
convert.bed2exons, 8
convert.bed2exons,data.frame-method (convert.bed2exons), 8
convert.bed2introns, 8
convert.bed2introns,data.frame-method (convert.bed2introns), 8

diffMethPerChr, 9
diffMethPerChr,methylDiff-method (diffMethPerChr), 9

filterByCoverage, 9
filterByCoverage,methylRaw-method (filterByCoverage), 9
filterByCoverage,methylRawList-method (filterByCoverage), 9

get.methylDiff, 10
get.methylDiff,methylDiff-method (get.methylDiff), 10
getAssembly, 11
getAssembly,methylBase-method (getAssembly), 11
getAssembly,methylRaw-method (getAssembly), 11
getAssociationWithTSS, 11
getAssociationWithTSS,-methods (getAssociationWithTSS), 11
getAssociationWithTSS,annotationByGenicParts-method (getAssociationWithTSS), 11
getContext, 11
getContext,methylBase-method (getContext), 11
getContext,methylDiff-method (getContext), 11
getContext,methylRaw-method (getContext), 11
getCorrelation, 12
getCorrelation,-methods (getCorrelation), 12
getCorrelation,methylBase-method (getCorrelation), 12
getCorrelation-method (getCorrelation), 12
getCoverageStats, 12
getCoverageStats,-methods (getCoverageStats), 12
getCoverageStats,methylRaw-method (getCoverageStats), 12
getData, 13
getData,-methods (getData), 13
getData,methylBase-method (getData), 13
getData,methylDiff-method (getData), 13
getData,methylRaw-method (getData), 13
getData-method (getData), 13
getFeatsWithTargetsStats, 13

getFeatsWithTargetsStats,annotationByFeature-method
(getFeatsWithTargetsStats), 13
getFlanks, 14
getFlanks,GRanges-method (getFlanks), 14
getMembers, 14
getMembers,annotationByFeature-method
(getMembers), 14
getMethylationStats, 15
getMethylationStats,methylRaw-method
(getMethylationStats), 15
getTargetAnnotationStats, 15
getTargetAnnotationStats,annotationByFeature-method
(getTargetAnnotationStats), 15
GRanges, *7, 8*, *22*
GRangesList, *25*

methylBase-class, 16
methylBase.obj, 16
methylDiff-class, 17
methylDiff.obj, 17
methylRaw-class, 17
methylRawList-class, 18
methylRawList.obj, 18

normalizeCoverage, 18
normalizeCoverage,methylRawList-method
(normalizeCoverage), 18

PCASamples, 19
PCASamples,methylBase-method
(PCASamples), 19
percMethylation, 20
percMethylation,methylBase-method
(percMethylation), 20
plotTargetAnnotation, 20
plotTargetAnnotation,annotationByFeature-method
(plotTargetAnnotation), 20
pool, 21
pool,methylBase-method (pool), 21

read, 21
read,character,character,character-method
(read), 21
read,list,list,character-method (read),
21
read.bed, 22
read.bed,character-method (read.bed), 22
read.bismark, 23
read.bismark,character,character,character-method
(read.bismark), 23
read.bismark,list,list,character-method
(read.bismark), 23
read.feature.flank, 24
read.feature.flank,character-method
(read.feature.flank), 24
read.transcript.features, 25
read.transcript.features,character-method
(read.transcript.features), 25
regionCounts, 25
regionCounts,methylRaw,GRanges-method
(regionCounts), 25
regionCounts,methylRaw,GRangesList-method
(regionCounts), 25
regionCounts,methylRawList,GRanges-method
(regionCounts), 25
regionCounts,methylRawList,GRangesList-method
(regionCounts), 25
reorganize, 26
reorganize,methylBase-method
(reorganize), 26
reorganize,methylRawList-method
(reorganize), 26

select, 27
select,methylBase-method (select), 27
select,methylDiff-method (select), 27
select,methylRaw-method (select), 27
show, 27
show,annotationByFeature-method (show),
27
show,annotationByGenicParts-method
(show), 27
show,methylDiff-method (show), 27

tileMethylCounts, 27
tileMethylCounts,methylRaw-method
(tileMethylCounts), 27
tileMethylCounts,methylRawList-method
(tileMethylCounts), 27

unite, 28
unite,-methods (unite), 28
unite,methylRawList-method (unite), 28
